# Structural snapshots of human PepT1 and PepT2 reveal mechanistic insights into substrate and drug transport across epithelial membranes

**DOI:** 10.1126/sciadv.abk3259

**Published:** 2021-11-03

**Authors:** Maxime Killer, Jiri Wald, Joanna Pieprzyk, Thomas C. Marlovits, Christian Löw

**Affiliations:** 1 Centre for Structural Systems Biology (CSSB), Notkestrasse 85, D-22607 Hamburg, Germany.; 2 European Molecular Biology Laboratory (EMBL), Hamburg Unit c/o Deutsches Elektronen Synchrotron (DESY), Notkestrasse 85, D-22607 Hamburg, Germany.; 3 Collaboration for joint PhD degree between EMBL and Heidelberg University, Faculty of Biosciences, Faculty of Biosciences, Im Neuenheimer Feld 234, D-69120 Heidelberg, Germany.; 4 Institute of Structural and Systems Biology, University Medical Center Hamburg-Eppendorf, Notkestrasse 85, D-22607 Hamburg, Germany.; 5 Deutsches Elektronen Synchrotron (DESY), Notkestrasse 85, D-22607 Hamburg, Germany.

## Abstract

The uptake of peptides in mammals plays a crucial role in nutrition and inflammatory diseases. This process is mediated by promiscuous transporters of the solute carrier family 15, which form part of the major facilitator superfamily. Besides the uptake of short peptides, peptide transporter 1 (PepT1) is a highly abundant drug transporter in the intestine and represents a major route for oral drug delivery. PepT2 also allows renal drug reabsorption from ultrafiltration and brain-to-blood efflux of neurotoxic compounds. Here, we present cryogenic electron microscopy (cryo-EM) structures of human PepT1 and PepT2 captured in four different states throughout the transport cycle. The structures reveal the architecture of human peptide transporters and provide mechanistic insights into substrate recognition and conformational transitions during transport. This may support future drug design efforts to increase the bioavailability of different drugs in the human body.

## Teaser

Structures of PepT1 and PepT2 provide mechanistic insights into substrate and drug translocation across epithelial membranes.

## INTRODUCTION

The plasma membrane forms a natural barrier for amino acids, short peptides, and other hydrophilic or charged nutrients. To preserve the distinct intracellular milieu, a large number of membrane transporters for these molecules have emerged during evolution to maintain the nutrient homeostasis of cells. For efficient uptake of individual amino acids and small peptides, specific amino acid transporters together with the promiscuous peptide transporter 1 (PepT1) are expressed in the mucosa of the small intestine (*1*). PepT1 belongs to the solute carrier family 15 (SLC15), also known as the proton-coupled oligopeptide transporter (POT) family, which consists of four members in eukaryotes: PepT1 (SLC15A1), PepT2 (SLC15A2), PhT1 (SLC15A4), and PhT2 (SLC15A3). PepT1 and PepT2 are best characterized and mediate the uptake, distribution, and resorption of di- and tripeptides in the body. These transporters are highly promiscuous and accept almost any di- and tripeptide, independent of their side-chain composition, but with substantial differences in affinity (*2*–*6*). PepT1 is the predominant paralog in the apical membrane of the intestinal epithelial cells, while PepT2 has a broad expression pattern and is mainly found not only in the kidney but also in various other tissues including the brain, neurons, lung, and choroid plexus (*7*–*10*).

PepT1 and PepT2 are secondary active transporters, which are energized by the inward-directed electrochemical proton gradient. This provides a driving force for transport and accumulation of nutrients above extracellular concentrations (*11*, *12*). Besides natural di- and tripeptides, PepT1 and PepT2 recognize and transport chemically diverse drug molecules such as β-lactam antibiotics, angiotensin-converting enzyme inhibitors, and antiviral drugs, thus affecting their availability, clearance, and distribution in the body (*13*–*19*). PepT1 accounts for ~50% of all known clinically relevant drug transporters in the small intestine and represents one of the main route for oral drug absorption (*20*). PepT2 reduces the clearance of exogenous molecules via renal tubular reabsorption (*21*) and enables drug efflux from the cerebrospinal fluid to the choroid plexus, thus influencing drug disposition, dynamics, and toxicity in the brain (*22*–*24*). In human disease, colonic expression of PepT1 leads to bacterial di- and tripeptide uptake in epithelial cells, causing downstream chronic inflammation and is associated with numerous gastrointestinal tract disorders including inflammatory bowel disease (IBD) and colonic cancer (*25*, *26*). PepT1-mediated uptake of tripeptides has been shown to reduce nuclear factor κB (NF-κB) and mitogen-activated protein (MAP) kinase inflammatory signaling pathways and proinflammatory cytokine secretion and reduced the incidence of colitis in mice, raising the use of anti-inflammatory oligopeptides as attractive therapeutic strategy against IBD (*27*–*30*).

On a structural level, human PepT1 (*Hs*PepT1) is 708 and human PepT2 (*Hs*PepT2) is 729 amino acids long. Both polypeptides consist of a core transporter unit of predicted 12 transmembrane helices (TMs) of the major facilitator superfamily (MFS) fold and an extracellular immunoglobulin-like domain (ECD) placed between TM9 and TM10. *Hs*PepT1 and *Hs*PepT2 share overall high sequence similarity (>70%), which is even higher for the transporter core units (>85% sequence similarity, ~65% sequence identity), and a highly conserved substrate binding site (fig. S1). Currently, it is postulated that substrate transport occurs via the “rocker-switch” alternating access mechanism, which involves conformational transitions between at least three different states: (i) outward open, (ii) occluded, and (iii) inward open (*31*).

Structures of different bacterial POT homologs have been determined over the past 10 years in apo, peptide-bound, and drug-bound states, exclusively representing inward-facing open or inward-facing partially occluded structures (*32*–*45*). This strongly limits our molecular understanding of the conformational transitions, required to complete an entire transport cycle. In particular, it is not clear whether the available inward-facing structures of the binding site are representative of the outward-facing state, where substrate recognition occurs.

Here, we present multiple cryogenic electron microscopy (cryo-EM) structures of *Hs*PepT1 and *Hs*PepT2 representing different stages of the transport cycle of the SLC15 family. *Hs*PepT1 was captured in the outward-facing conformation, both in apo and substrate-bound states, and in a substrate-bound outward-facing occluded structure. *Hs*PepT2, on the other hand, has been trapped in a substrate-bound inward-facing partially occluded state. This work reveals the architecture of human POTs, which differ from the bacterial homologs, and elucidates substrate coordination in the centrally located binding cavity. Because of the availability of different conformational states of these highly similar transporters, we obtained molecular insights in conformational changes occurring during the entire transport cycle required for substrate recognition and transport. Our work will form the basis for future drug design and modification approaches using peptide transporters as shuttle systems.

## RESULTS

### Expression, purification, and structure determination of *Hs*PepT1 and *Hs*PepT2

**Fig. 1.**
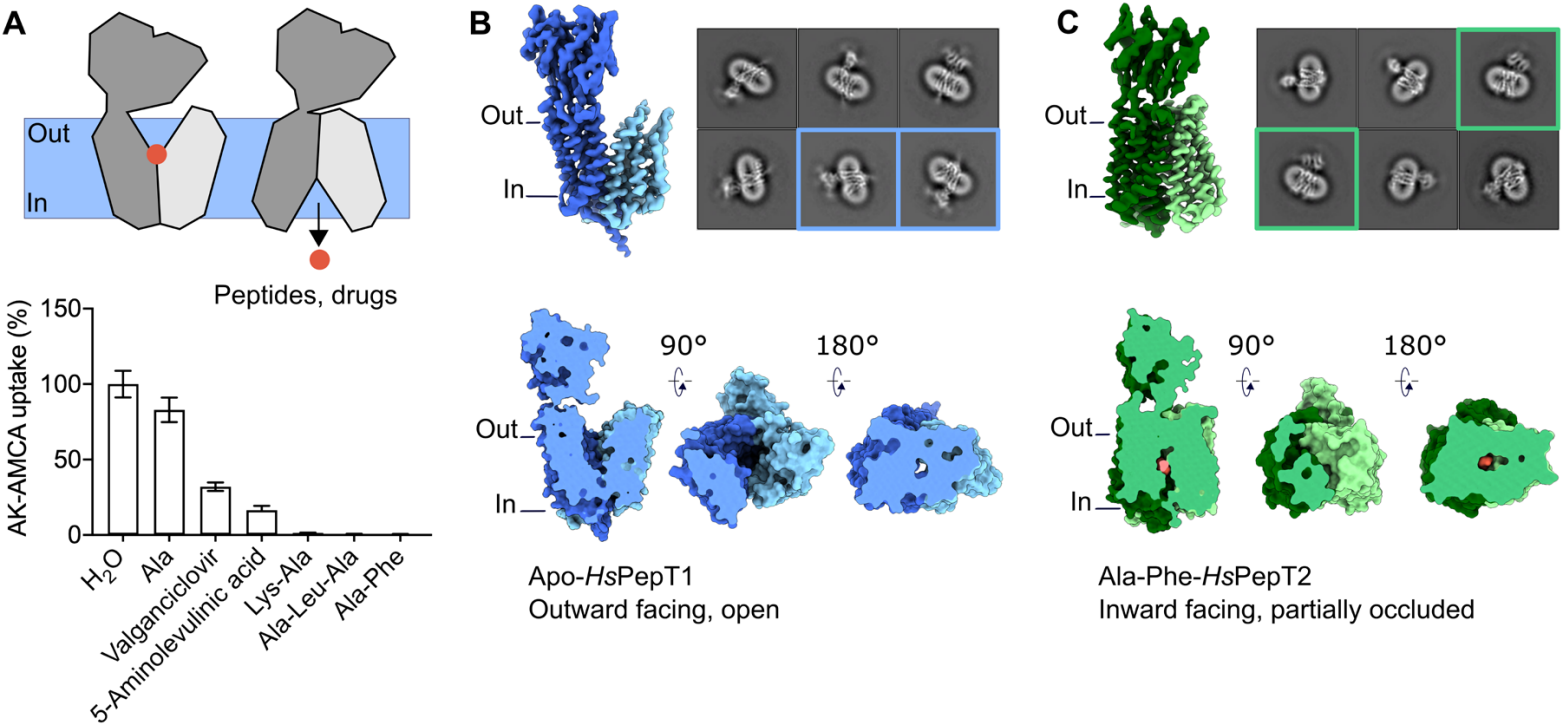
Cryo-EM structures of apo *Hs*PepT1 and *Hs*PepT2 bound to Ala-Phe. (**A**) Whole-cell transport competition assays of the β-Ala-Lys peptide coupled to the fluorescent AMCA moiety (AK-AMCA) in *Hs*PepT2-transfected HEK293 cells showing reduced AK-AMCA uptake in the presence of 5 mM of the competing substrate. (**B** and **C**) Three-dimensional reconstructions of (B) *Hs*PepT1 and (C) *Hs*PepT2 with corresponding 2D class averages and surface representation highlighting the (B) outward-facing open and (C) inward-facing partially occluded conformations.

*Hs*PepT1 and *Hs*PepT2 were expressed in human embryonic kidney (HEK) 293 cells. To monitor transport, we made use of the fluorescently labeled dipeptide (ß-Ala-Lys-AMCA) (*46*) and confirmed specific uptake of this compound after *Hs*PepT2 overexpression (Fig. 1A and fig. S2). Different di- and tripeptides efficiently compete for the same binding site resulting in reduced uptake of the fluorescent reporter. The same expression system has been used for *Hs*PepT1 transport assays in the past (*47*). Initially, we reconstituted *Hs*PepT2 in saposin-lipid nanoparticles to better mimic a lipid bilayer (*48*, *49*). This approach yielded a homogeneous sample, but after grid preparation and imaging, it became clear that the particles adopted a preferred orientation on the cryo-EM grid (mainly top views) resulting in a poorly interpretable volume (fig. S3, C to F). Therefore, we imaged monomeric full-length *Hs*PepT1 and *Hs*PepT2 extracted in detergent (Fig. 1 and fig. S3). In a first step, the structure of *Hs*PepT1 was investigated in its apo form, while *Hs*PepT2 was preincubated with one of its natural substrates. Distinct conformational states were already noticeable from two-dimensional (2D) class averages between the two paralogs, with *Hs*PepT1 representing an outward-facing conformation and *Hs*PepT2 representing an inward-facing state. We obtained 3D reconstructions for apo *Hs*PepT1 at a nominal resolution of 3.9 Å (fig. S4) and for *Hs*PepT2 bound to the dipeptide Ala-Phe at a nominal resolution of 3.8 Å with an estimated local resolution of up to 3.2 Å within the transporter unit (fig. S5 and table S1).

### Architecture of human peptide transporters

**Fig. 2.**
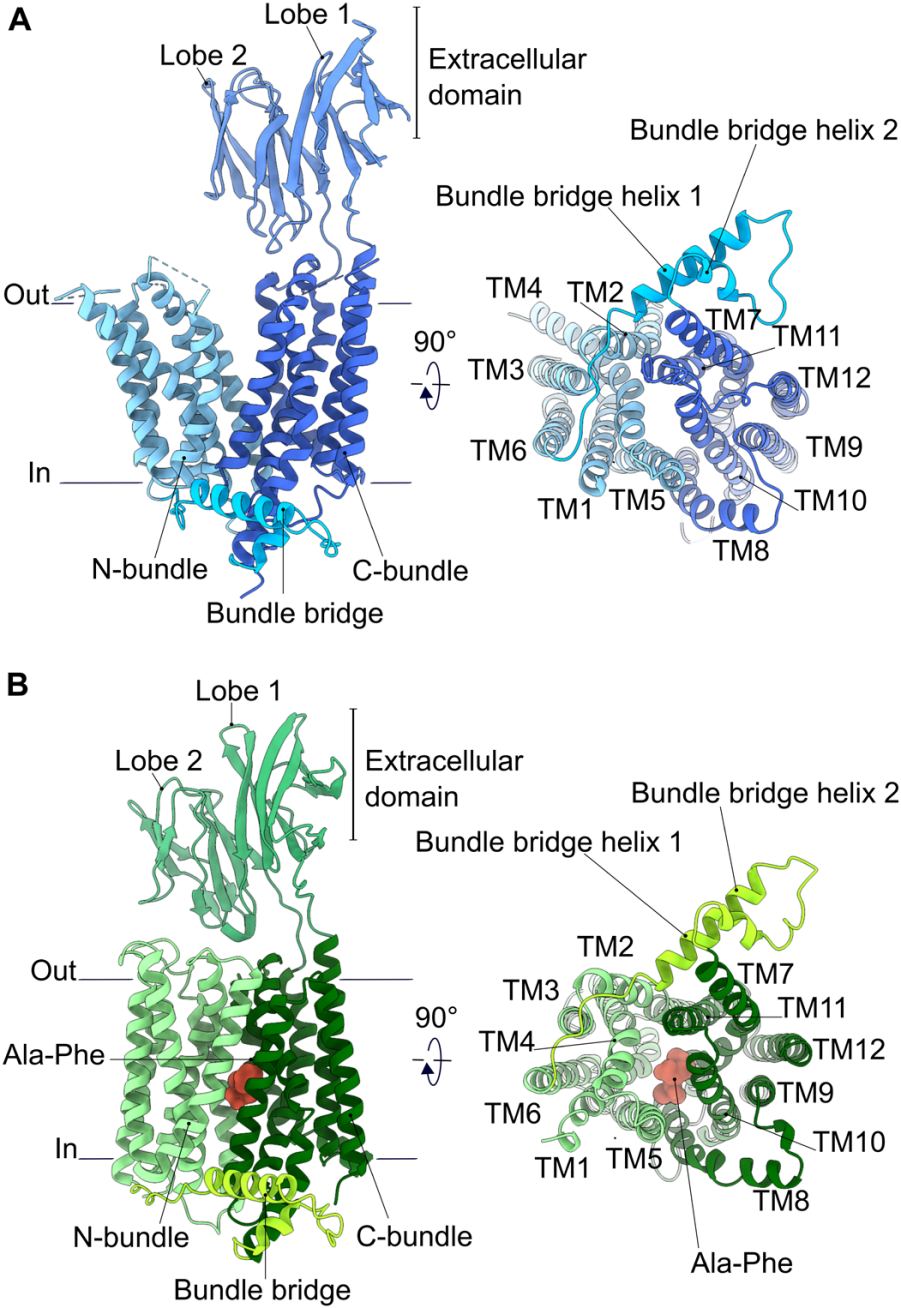
Overall architecture of human POTs. (**A**) Apo-*Hs*PepT1 and (**B**) substrate-bound *Hs*PepT2 models shown as cartoon representation. The different architectural elements are labeled. Loops that could not be modeled because of poor density are shown as dashed lines.

The 3D reconstructions allowed de novo modeling of most of the MFS transporter units, which consist of 12 TMs and a long linker connecting both helical bundles (figs. S6 and S7). Concerning the *Hs*PepT1 structure, 11 residues at the N terminus, four loops connecting TM1-TM2 (residue 49), TM3-TM4 (residues 109 to 116), TM5-TM6 (residues 189 to 194), and TM11-TM12 (residues 638 to 641), and the last 25 residues at the C terminus could not be modeled because of poor density likely caused by intrinsic dynamics and partial disorder. The *Hs*PepT2 model is lacking the first 40 residues at the N terminus and the last 32 C-terminal residues, which are predicted to be disordered. The transmembrane domains of *Hs*PepT1 and *Hs*PepT2 adopt the canonical MFS fold formed by 12 TMs organized in two six-helix bundles with both N and C termini facing the cytoplasm (Fig. 2). The bundles are connected via a long cytoplasmic linker, which encompasses two α helices that interact with each other. This linker, which we have termed the “bundle bridge,” is likely common for all SLC15 transporters (figs. S1 and S8). The bundle bridge is of amphipathic nature and is associated with the inner leaflet of the plasma membrane (fig. S8). Its role is currently unclear, but it might take part in lipid sensing or in providing a platform for other interacting proteins.

Within the MFS, mammalian PepT1 and PepT2 are the only known transporters with an additional ECD placed between TM9 and TM10. As the extracellular domain of *Hs*PepT2 was poorly resolved, the predicted structure from AlphaFold (*50*) was used for template-based model refinement in this region. The higher local resolution in *Hs*PepT1-ECD allowed us to identify six N-linked glycans (fig. S9), five of which were experimentally confirmed to be present on murine PepT1 and are likely involved in protein folding, membrane targeting, and protection from proteolytic degradation (*51*). In the context of the full-length transporter, the arrangement of the two immunoglobulin lobes in *Hs*PepT1 differs strongly from the previously crystallized isolated murine soluble PepT1-ECD (*52*) but is very similar to *Hs*PepT2-ECD. Although *Hs*PepT1 and *Hs*PepT2 display different conformational states, both extracellular domains are positioned similarly with respect to the C-terminal bundle, with small hinge movements in relation to the linker region, and no interaction is observed between the ECDs and the N-bundle. This observation is in agreement with previous work, highlighting that the ECD is not essential for substrate transport but potentially forms an interaction platform for proteases such as trypsin via a conserved acidic motif (*52*). Nevertheless, it cannot be excluded that the presence of a lipid environment might have an influence on the position of the ECD with respect to the transporter unit. The overall structures of *Hs*PepT1 and *Hs*PepT2 reveal notable architectural differences between human and bacterial homologs, as illustrated in fig. S10. While *Hs*PepT1 and *Hs*PepT2 contain 12 TMs, an additional ECD, and the bundle bridge connecting the N- and C-terminal bundles, bacterial POT structures display two additional TMs (HA-HB) of currently unknown function and lack the soluble domain (*32*).

### Major conformational changes between inward- and outward-facing states

**Fig. 3.**
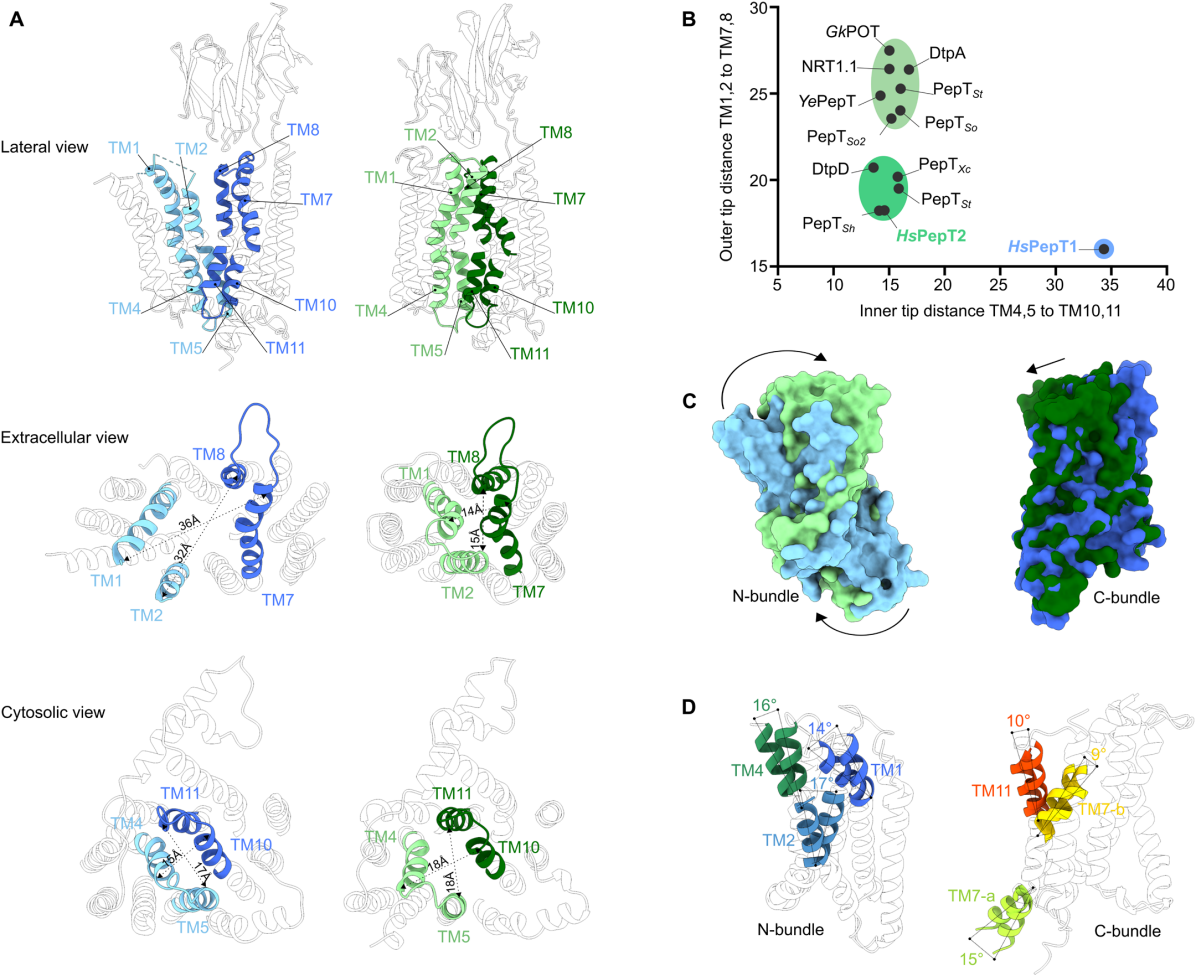
Structural comparison between the outward- and inward-facing states observed in apo *Hs*PepT1 and substrate-bound *Hs*PepT2. (**A**) Opening and closing of the substrate binding site to the extracellular and intracellular milieu observed in *Hs*PepT1 (blue) and *Hs*PepT2 (green). (**B**) The distances between C_α_ atoms of the relevant pairs of helix tips from all bacterial POTs determined by x-ray crystallography were measured and compared to the human transporters. (**C**) Rocking motions of the N-bundle (*Hs*PepT1: light blue and *Hs*PepT2: light green) and C-bundle (*Hs*PepT1: dark blue and *Hs*PepT2: dark green) after structural alignment of both transporter units. (**D**) Bending of TMs with measured tilt angles observed in the N-bundle (left) and C-bundle (right) between *Hs*PepT1 and *Hs*PepT2.

In the outward open state of apo *Hs*PepT1, the central substrate binding cavity of the transporter unit is widely exposed to solute molecules from the extracellular space with an opening of approximately 30 Å (Fig. 3A). So far, among the available POT structures, no outward-facing conformation has been observed (Fig. 3B). This previously not captured state allowed us to observe the major structural changes occurring between outward- and inward-facing conformations. As the sequence conservation between *Hs*PepT1 and *Hs*PepT2 is high (>70% similarity), we superimposed their transmembrane domains to highlight the differences between these two states. The transition from *Hs*PepT1 outward-facing conformation to the inward-facing state observed in *Hs*PepT2 can be deconvoluted in two main processes: (i) a large rocking motion of the N-bundle coupled with a smaller movement of the C-bundle, carrying along the extracellular domain (Fig. 3C), and (ii) an additional bending of TM1, TM2, TM4, TM7, and TM11, allowing for a further opening and closing of the cytoplasmic and extracellular side, respectively (Fig. 3D).

In the *Hs*PepT2 structure, the cytoplasmic side ends of TM4 and TM5 in the N-bundle are in close proximity to TM10 and TM11 in the C-bundle, narrowing the exit route from the peptide binding site to the cytosol, although without fully closing it as instead was observed in outward-facing *Hs*PepT1 (Figs. 1C and 3A). Such a conformation is referred to as “partially occluded.” As previously shown in bacterial homologs studies, the transition from fully occluded to fully inward open conformation occurs via bending of TM10 and TM11 toward TM4 and TM5 (fig. S11) (*34*, *39*, *41*, *42*). Upon switching to the outward-facing conformation, the modest rocking movement of the C-bundle translates the TM10-TM11 hairpin in closer proximity to TM4-TM5, resulting in tight sealing of the substrate binding pocket from the cytosol (Fig. 3A).

**Fig. 4.**
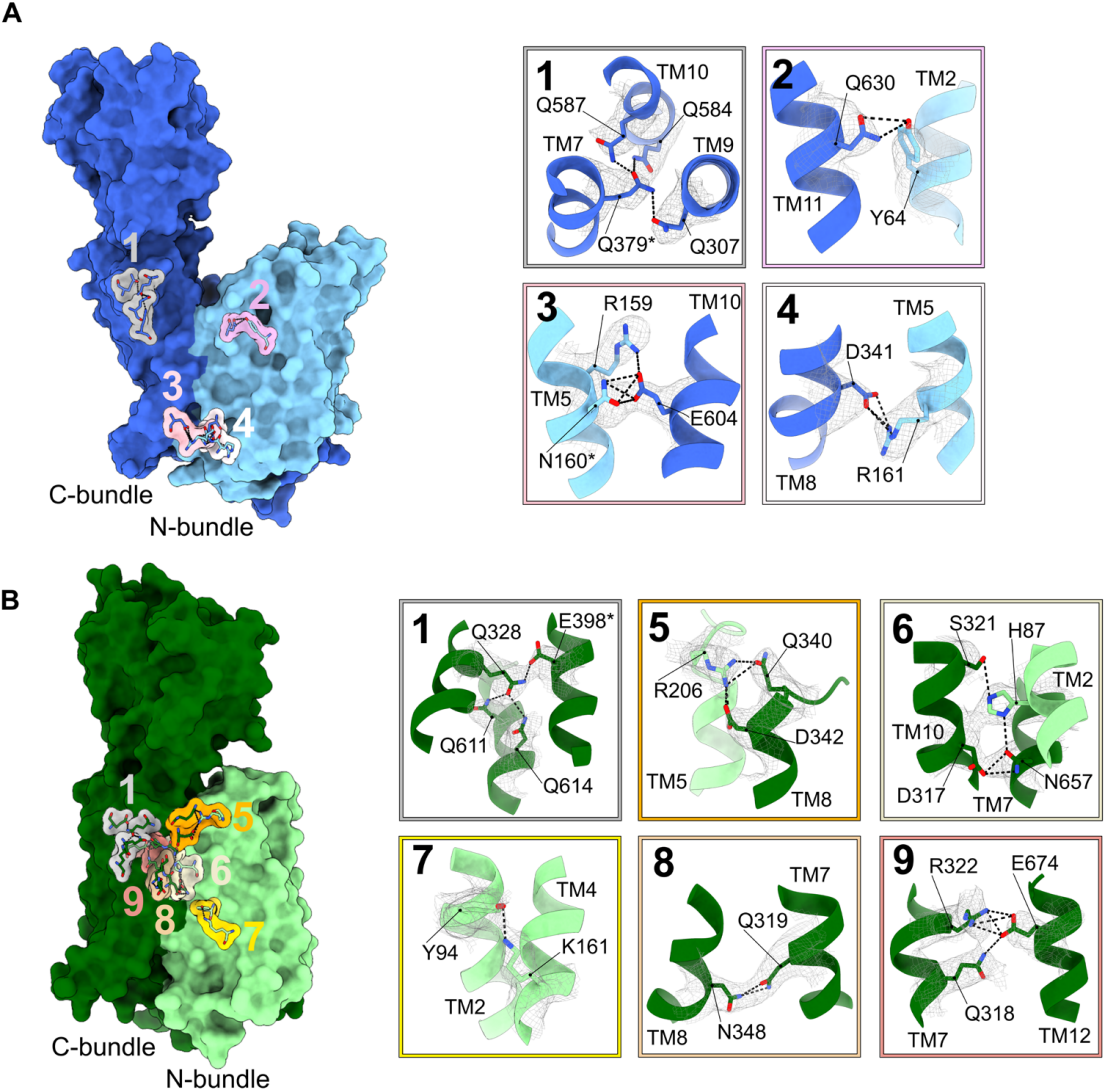
Interactions stabilizing the outward-facing open state of *Hs*PepT1 and the inward-facing partially occluded state of *Hs*PepT2. The locations of key interactions are shown and labelled on (**A**) *Hs*PepT1 and (**B**) *Hs*PepT2. Corresponding close-up views show the cryo-EM densities of the side chains forming the interactions as indicated by dashed lines.

While a network of glutamine residues in TM7, TM9, and TM10 restricts conformational changes within the C-bundle in proximity to the extracellular domain in both *Hs*PepT1 and *Hs*PepT2, other interactions between polar residues conserved among mammalian PepT1 and PepT2 are formed and broken between outward- and inward-facing conformations (Fig. 4 and fig. S1). In *Hs*PepT1, the large bending of TM2 is stabilized by a hydrogen bond between Y64 (TM2) and N630 (TM11) in the extracellular region of the transporter unit. The sealing of the cytosolic side is stabilized by two interbundle salt bridges between R159 (TM5)–E604 (TM10) and R161 (TM5)–D341 (TM8) (Fig. 4A).

In the partially occluded inward-facing state of *Hs*PepT2, these interbundle salt bridges on the cytosolic side are disrupted (Fig. 4B), allowing the large rocking movement of the N-bundle (Fig. 3C). In the upper part of the transporter unit, the interaction between Y94 in TM2 (Y64 in *Hs*PepT1) and N657 (N630 in *Hs*PepT1) is disrupted and replaced by an interaction between Y94 and K161 (TM4), restricting bending of TM2. Additional intrabundle contacts including Q319 (TM7)–N348 (TM8) and Q316–R322 (TM7)–E674 (TM12) further stabilize the partially occluded inward-facing state. Last, the interbundle salt bridge between R206 (TM5) and D342 (TM8), together with the polar interaction network around H87 involving TM2, TM7, and TM8, tightly seals the central cavity from the extracellular space. These interactions need to be disrupted so that the transporter can cycle back to the outward-facing state observed in *Hs*PepT1. The interaction networks are illustrated in Fig. 4 (A and B). Mutational studies on *Hs*PepT1 identified H57 (H87 *Hs*PepT2) as a critical residue for substrate translocation in both transporters (*53*), in agreement with our data presented here. This residue, as well as four of the five interbundle interaction networks mentioned above, is conserved in mammalian POTs, but not in bacterial homologs (fig. S1). This might indicate an evolutionary divergence in the mechanism of conformational changes throughout the transport cycle of the POT family.

### Dynamics in the transport cycle induced upon substrate binding

**Fig. 5.**
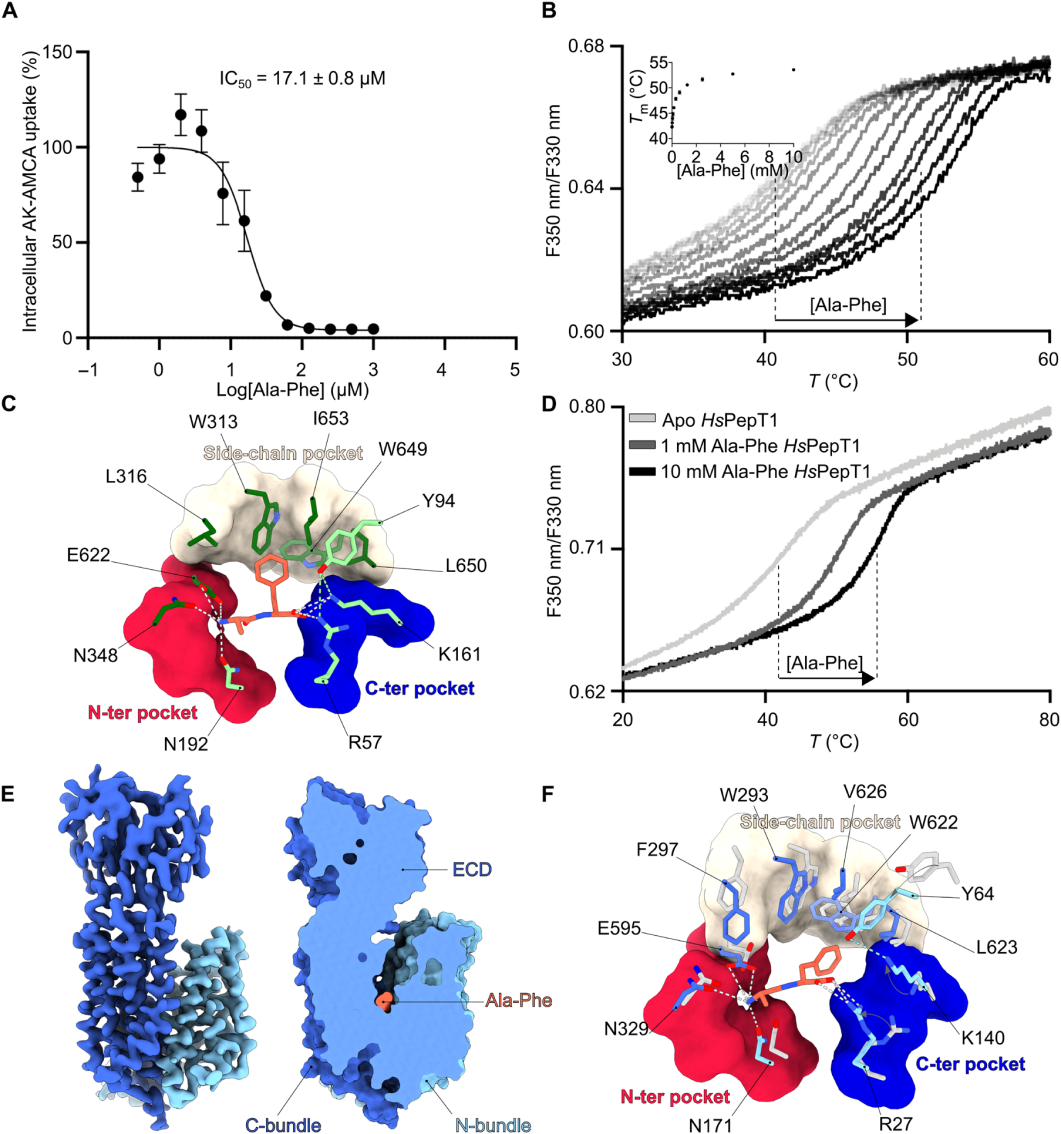
Structural basis for substrate recognition in human POTs. (**A**) Concentration-dependent competition assay of the β-Ala-Lys peptide coupled to the fluorescent AMCA moiety (AK-AMCA) in *Hs*PepT2 with the dipeptide Ala-Phe. The average uptake value for each condition was calculated from three independent measurements. The error bars correspond to the SD from these independent measurements. (**B**) Thermal stabilization of detergent-solubilized *Hs*PepT2 upon substrate binding measured by nano-differential scanning fluorimetry (DSF) at increasing concentrations of Ala-Phe (inset shows the increase in melting temperature with increasing peptide concentration). (**C**) Close-up view of the *Hs*PepT2 peptide binding site. Electrostatic interactions between the peptide (shown in orange) and the transporter (shown in green) are displayed as gray dashes. The interaction between Y94 and the C-ter pocket is shown as green dashes. The different pockets are indicated. (**D**) Thermal stabilization of detergent solubilized *Hs*PepT1 upon substrate binding measured by nano-DSF at increasing concentrations of Ala-Phe. (**E**) Three-dimensional reconstruction of outward-facing open *Hs*PepT1 bound to Ala-Phe (**F**) Overlay of the binding sites of outward-facing open apo (shown in gray) and Ala-Phe–bound *Hs*PepT1 (shown in blue colors). Electrostatic interactions between the peptide (shown in orange) and the transporter are displayed as gray dashes. The interaction between Y64 and the C-ter pocket is shown as blue dashes.

Initially, we determined the structures of *Hs*PepT1 in the absence of a substrate and of *Hs*PepT2 in the presence of the dipeptide Ala-Phe. We used the fluorescently labeled dipeptide ß-Ala-Lys-AMCA as a transport reporter in cell-based uptake assays to confirm binding of the dipeptide Ala-Phe, other peptides, and known drugs to *Hs*PepT2. Concentration-dependent competition experiments yielded median inhibitory concentration (IC_50_) values in the micromolar range, with naturally occurring substrates displaying higher affinities (IC_50_ = 17.1 ± 0.8 μM to 45.5 ± 0.1 μM) compared to the tested known transported drugs, such as valganciclovir or 5-aminolevulinic acid (IC_50_ = 368.9 ± 1.5 μM and 373.5 ± 1.6 μM) (Fig. 5A and fig. S2). Recombinantly expressed and purified *Hs*PepT2 used for structure determination was stabilized against heat unfolding in the presence of the dipeptide Ala-Phe in a concentration-dependent manner, confirming substrate binding of detergent extracted *Hs*PepT2 (Fig. 5B). The higher local resolution in the substrate binding site of *Hs*PepT2 enabled us to unambiguously assign the extra density to the dipeptide Ala-Phe and model all coordinating *Hs*PepT2 side chains (Fig. 5C and fig. S12A). The N and C termini of the dipeptide are clamped by electrostatic interactions with N192, N348, E622, R57, and K161, respectively. The phenyl group side chain is accommodated in a hydrophobic pocket formed by Y94, W313, L316, W649, L650, and I653, referred to as the side-chain pocket. The interactions between the peptide backbone and *Hs*PepT2 are in agreement with previous biochemical and mutational studies in mammalian POTs (*13*, *53*, *54*) and structural studies on inward-facing open or partially occluded states of bacterial homologs (*33*, *34*).

Subsequently, to assess whether the switch from the outward-facing open state (as observed in the apo *Hs*PepT1 structure) to the inward-facing partially occluded state (observed in the Ala-Phe–bound *Hs*PepT2 structure) had been triggered by substrate binding, we also imaged and determined the structure of *Hs*PepT1 in the presence of the same dipeptide, which also stabilized the transporter against heat unfolding in a concentration-dependent manner (Fig. 5D). However, even in the presence of the Ala-Phe dipeptide, the conformation of *Hs*PepT1 remained outward facing. Nevertheless, this time, we could differentiate between two distinct conformations in the same dataset, both different from the apo outward-facing open state (figs. S13 and S14). The data resulted in a lower resolution reconstruction for the substrate-bound outward-facing occluded state (nominal resolution of 4.1 Å) and a substrate-bound outward-facing open state at a nominal resolution of 3.5 Å, with an estimated local resolution of up to 3.0 Å within the transporter unit (Fig. 5E and figs. S13 and S15). This allowed us to compare the binding sites of outward-facing open *Hs*PepT1 in its apo and substrate-bound forms (Fig. 5F). The structural overlay reveals that the negatively charged N-terminal pocket of the binding site is already positioned to coordinate the C terminus of the substrate in the central cavity. On the other hand, the positively charged C-terminal pocket undergoes larger rearrangements upon substrate binding, resulting in tightening of the central cavity around the substrate (fig. S16). Notably, the N-terminal pocket consists of residues located in the C-bundle of the transporter, while the more flexible C-terminal pocket comprises residues from the N-bundle, in agreement with the dynamic observations made between outward- and inward-facing *Hs*PepT1 and *Hs*PepT2.

**Fig. 6.**
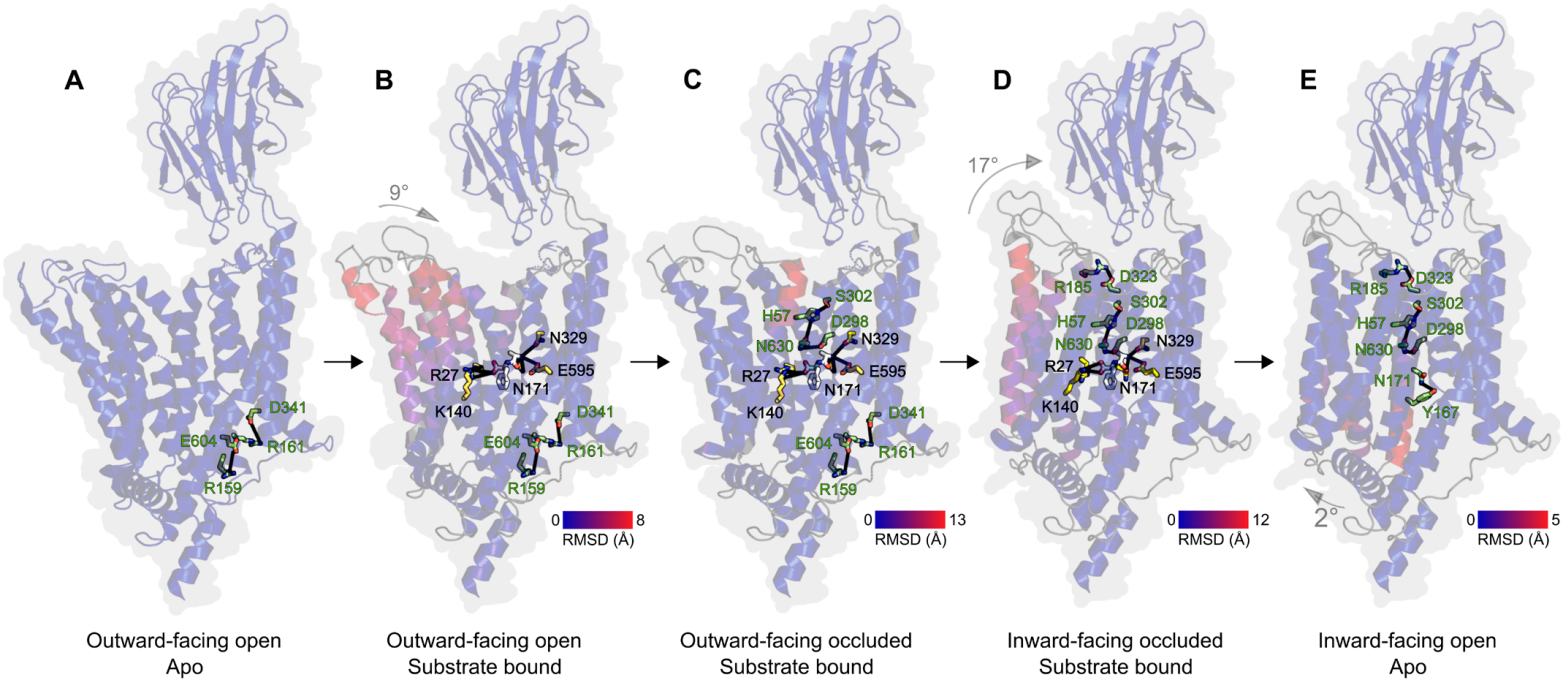
Mechanism for substrate recognition and transport in human POTs based on the presented structures. (**A**) In the first step of the transport cycle, the transporter is in an outward-facing open state stabilized by two salt bridges between R159-E604 and R161-D341. (**B**) Upon peptide binding—accommodated in the charged central cavity via its N terminus by N171, N329, and E595 and optionally its C terminus by R27 and K140—the N-bundle helices follow bending and rigid body motions resulting in tightening of the central cavity. (**C**) Further bending of TM2 allows the interaction of H57, S302, N630, and D298 as a crucial step before (**D**) total sealing of the extracellular side and switching to the inward-facing occluded state stabilized by the salt bridge R185-D323. (**E**) Last, opening of the cytosolic side is achieved by TM4 and TM5 moving away from TM10 and TM11 resulting in the loss of the crucial interaction between the transporter and the peptide termini, allowing substrate release to the cytoplasm. The structures shown in (A), (B), and (C), represent models of *Hs*PepT1 on the basis of the three different cryo-EM maps presented in this article. Missing loops have been added. The structure in (D) is derived from the experimental *Hs*PepT2 structure. (E) corresponds to the AlphaFold structure prediction (*50*) available in the EMBL-EBI AlphaFold database. Numbering of residues illustrated in this model follow the *Hs*PepT1 nomenclature. Conformational changes along the reaction cycle are colored according to the root mean square deviation (RMSD) between different state structures.

These additional structures, which represent intermediate states between the outward- and inward-facing states described above, allow us to finally get a more detailed, step-by-step understanding of the molecular events required for transport and mediated by rearrangements of the N-bundle (Fig. 6 and movie S1). Upon peptide binding, the tightening of the central cavity described above is caused by small bending movements of the N-bundle helices and a 9° rocking motion of the entire N-bundle toward the C-bundle (“outward-facing open substrate bound”). This is then followed by a large bending tilt of 34° of TM2, resulting in the occlusion of the extracellular side, stabilized by the interaction of H57, S302, N630, and D298 (“outward-facing occluded substrate bound”). This interaction network requires protonation of H57 and facilitates the large transition to the inward-facing partially occluded conformation observed in *Hs*PepT2, which can be dissected in helix bending motions and a large 17° rocking motion of the whole N-bundle (“inward-facing occluded substrate bound”). In this transition, salt bridges present on the intracellular side of all previous outward-facing states are disrupted, and a new one is established on the extracellular side, leading to the complete sealing from the luminal solvent. Last, the AlphaFold structure predictions (*50*) of apo *Hs*PepT1 and apo *Hs*PepT2 in their inward-facing open states suggest that opening of the cytosolic side is mediated via a movement of TM4-TM5 away from the TM10-TM11 hairpin, resulting in the loss of crucial interactions between the transporter and the peptide termini (fig. S14C) and substrate release into the cytoplasm (“inward-facing open apo”).

## DISCUSSION

This work defines the architecture of the human peptide transporters *Hs*PepT1 and *Hs*PepT2, describes the associated conformational changes during a transport cycle in molecular detail, and illustrates how substrates are coordinated in the binding pocket within these promiscuous transporter family, as summarized in Fig. 6. More than two decades of biochemistry work on this extensively studied protein family can now be placed in context of the available structures for *Hs*PepT1 and *Hs*PepT2. Structural and functional studies of bacterial POTs over the past years have helped in understanding how certain drugs are bound and how substrate promiscuity might be achieved. Nevertheless, all structures of bacterial homologs [47 Protein Data Bank (PDB) entries representing 10 different bacterial POTs] have been captured in the inward-facing open or inward-facing partially occluded state, limiting our understanding of the conformational changes during transport. To date, it is unclear why both human transporters display different conformational states under the measured conditions, because a recent single-molecule fluorescence resonance energy transfer study on the bacterial POT DtpA confirmed the inward-open state as the lowest energy state in detergent solution (*55*, *56*) in agreement with the deposited POT structures. Multiple stabilizing interactions, mainly salt bridges between the N- and C-terminal bundle, need to be broken and formed during a transport cycle (*31*, *57*). This work now highlights that most identified residues involved in interbundle interactions are not conserved throughout evolution among all phyla, and different residues contribute orchestrating the conformational changes of the TMs during transport. According to the proposed rocker-switch mechanism for MFS transporters, the N- and C-terminal bundles of the transporter unit mainly function as rigid bodies with a certain degree of bending motion to drive alternate access (*58*, *59*). Nevertheless, besides the rocking motions, we also observe strong and independent bending movements of multiple TMs, more in agreement with the proposed “clamp and switch model” for MFS transporters (*31*, *57*). A structural overlay of the N- and C-bundles of *Hs*PepT1 and *Hs*PepT2 reveals a stronger deviation for the N-bundle, indicating increased flexibility and dynamics in this part of the molecule, while the C-bundle remains rather rigid. This is in contrast to bacterial POTs (*39*, *41*, *42*) and sugar transporters (*31*, *57*), where the N-terminal bundle has been identified as the most stable and rigid part of the molecule. This might be caused by the required stabilizing function of the C-bundle for the additional extracellular domain located between TM9 and TM10 in *Hs*PepT1 and *Hs*PepT2. This is supported by the tight interaction network within the C-terminal bundle formed by the glutamine girdle (Fig. 4).

Our work created the framework for understanding the molecular transport mechanism of human POTs, which, in turn, will accelerate structure-based drug design approaches aiming to increase the bioavailability of different compounds in the human body via these transport systems. At the same time, despite available eukaryotic POT structures, the prediction of drug coordination remains challenging and additional transporter structures bound to a diverse set of drug or inhibitor molecules are required to obtain a more detailed understanding of drug recognition of this promiscuous transporter family.

## MATERIALS AND METHODS

### Expression and purification of *Hs*PepT2

The N528Q-N587Q *Hs*PepT2 gene was cloned into a pXLG vector containing an expression cassette composed of an N-terminal Twin-Streptavidin tag followed by the human rhinovirus 3C (HRV-3C) protease recognition sequence. The double mutation in *Hs*PepT2 was introduced to decrease sample heterogeneity caused by glycosylation and to increase expression levels. HEK293F cells were collected 48 hours after transient transfection as previously described (*60*) and stored at −80°C until further use. Frozen cell pellets were resuspended in 300 mM NaCl, 20 mM NaPi (pH 7.5), 0.5 mM tris(2-carboxyethyl)phosphine) (TCEP), and 5% glycerol, supplemented with cOmplete EDTA-free protease inhibitors, and were disrupted using an Avestin Emulsiflex homogenizer. The lysate was centrifuged for 10 min at 10,000*g*, and the supernatant was centrifuged for 90 min at 95,000*g* (Optima XE-90, Beckman Coulter). The pellet containing the membrane fraction was solubilized in 1% *N*-dodecyl-β-d-maltopyranoside (DDM; Anatrace) and 0.1% cholesteryl hemisuccinate (CHS; Tris Salt, Anatrace) for 1 hour at 4°C. The sample was centrifuged for 50 min at 70,000*g*, and the supernatant was applied to Strep-TactinXT beads (IBA). After 20 min of incubation on a rotating wheel, the suspension was transferred to a gravity column. Following two wash steps with 300 mM NaCl, 20 mM Hepes (pH 7.5), 0.03% DDM, and 0.003% CHS, *Hs*PepT2 was eluted with 0.03% DDM, 0.003% CHS, 150 mM NaCl, 20 mM Hepes (pH 7.5), and 10 mM desthiobiotin (Sigma-Aldrich). 3C cleavage was performed in 30 min, and the protease was separated from *Hs*PepT2 by gel filtration using Superose 6 Increase 10/300 (Sigma-Aldrich). The top fraction was concentrated to 10 mg/ml using a 100-kDa cutoff concentrator (Corning Spin-X UF concentrators) and stored at −80°C until further use.

### Expression and purification of *Hs*PepT1

The wild-type *Hs*PepT1 gene was cloned into a pXLG vector containing an expression cassette composed of an N-terminal Twin-Streptavidin tag followed by the HRV-3C protease recognition sequence. HEK293F cells were collected 48 hours after transient transfection and resuspended in 300 mM NaCl, 20 mM NaPi (pH 7.5), 0.5 mM TCEP, and 5% glycerol, supplemented with cOmplete EDTA-free protease inhibitors. Whole cells were solubilized overnight in 1% lauryl maltose neopentyl glycol (LMNG; Anatrace) and 0.2% CHS (Tris Salt Anatrace). The sample was then centrifuged for 60 min at 70,000*g*, and the supernatant was applied to Strep-TactinXT beads (IBA). After 30-min incubation on a rotating wheel, the suspension was transferred to a gravity column. Following two wash steps with 300 mM NaCl and 20 mM Hepes (pH 7.5) supplied with 0.03% DDM, 0.003% LMNG, and 0.006% CHS, *Hs*PepT1 was eluted with 0.03% DDM, 0.003% LMNG, 0.0006% CHS, 150 mM NaCl, 20 mM Hepes (pH 7.5), and 10 mM desthiobiotin (Sigma-Aldrich).

The sample was concentrated to 100 μl using a 100-kDa cutoff concentrator (Corning Spin-X UF concentrators) and run directly on a Superdex Increase 200 5/150 gel filtration column for vitrification in 0.015% DDM, 0.0015% LMNG, 0.003% CHS, 150 mM NaCl, 50 mM Hepes (pH 7.5), and 0.5 mM TCEP. The top fraction reached a concentration of 2 mg/ml and was stored at −80°C until further use.

### Whole-cell uptake assays

The wild-type *Hs*PepT2 gene was cloned into a pXLG vector (*61*) containing an expression cassette composed of an N-terminal hexa-histidine tag followed by enhanced green fluorescent protein and a tobacco etch virus protease cleavage site. HEK293F cells grown in suspension in FreeStyle medium were transfected with wild-type *Hs*PepT2 using a mass ratio of 2:1 polyethyleneimine:DNA. *Hs*PepT2 was expressed for 48 hours at 37°C, 8% CO_2_ at 220 rpm. For competition assays, 4 × 10^6^ cells/ml resuspended in phosphate-buffered saline buffer at pH 6.0 supplemented with 5 mM glucose were incubated in 96-well plates, with 50 μM β-Ala-Lys-AMCA in the absence or presence of dipeptides, tripeptides, or drugs for 10 min at 37°C. The reaction was stopped by adding 200 μl of ice-cold buffer, and the cells were then washed three times with the same buffer. Last, the cells were resuspended in 200 μl of buffer, and the fluorescence was measured in an M1000 microplate reader (TECAN) with excitation at 350 nm and emission at 450 nm. All experiments were performed in triplicates. The results were normalized by the fluorescence value of the control (cells overexpressing *Hs*PepT2 incubated with AK-AMCA in the absence of inhibitor) and plotted as AK-AMCA uptake rate percentage. For concentration-dependent uptake experiments, IC_50_ values were processed in GraphPad Prism 9.0 (GraphPad Software) using sigmoidal four-parameter curve fitting.

### Thermal stability measurements

The differential scanning fluorimetry method was used to follow the thermal unfolding event of *Hs*PepT2 and *Hs*PepT1 with a Prometheus NT.48 device (NanoTemper Technologies, Munich, Germany). Purified *Hs*PepT2 was diluted to 0.3 mg/ml and supplemented with decreasing amounts of Ala-Phe dipeptide in a dilution series of 13 points, starting at 10 mM down to 2.4 μM. Purified *Hs*PepT1 was diluted to 0.3 mg/ml and supplemented with 0, 1, or 10 mM Ala-Phe. The fluorescence at 330 and 350 nm was recorded over a temperature gradient scan from 15° to 95°C and processed in GraphPad Prism 9.0 (GraphPad Software).

### Cryo-EM sample preparation and data collection on apo *Hs*PepT1 in the outward-facing open state

Four microliters of purified *Hs*PepT1 at 2 mg/ml was applied to a glow-discharged gold holey carbon 2/1 300-mesh grid (Quantifoil). The grid was blotted for 4 s at 0 force before being plunge-vitrified in liquid propane using Mark IV Vitrobot (Thermo Fisher Scientific). The blotting chamber was maintained at 4°C and 100% humidity during freezing. Movies were collected using a Titan Krios (Thermo Fisher Scientific) equipped with a K3 camera and BioQuantum energy filter (Gatan) set to 20 eV. A total of 22,537 movies were collected at a nominal magnification of ×105,000 and a physical pixel size of 0.85 Å, with a 70-μm C2 aperture and 100-μm objective aperture at a dose rate of 16 e^−^/pixel per second. A total dose of 66 e^−^/Å^2^ was collected with 3-s exposure as movies of 50 frames. Data were collected using EPU (Thermo Fisher Scientific).

### Cryo-EM sample preparation and data collection on *Hs*PepT2 bound to Ala-Phe in the inward-facing partially occluded state

One hour before vitrification, purified N528Q-N587Q *Hs*PepT2 was thawed on ice and run on a Superdex Increase 200 5/150 column in 0.015% DDM, 0.0015% CHS, 100 mM NaCl, 10 mM Hepes (pH 7.5), and 0.5 mM TCEP. The top fraction reached a concentration of 1 mg/ml, and 3.6 μl supplemented with 5 mM of the dipeptide alanine-phenylalanine (Bachem) was applied to glow-discharged gold holey carbon 2/1 300-mesh grids (Quantifoil). Grids were blotted for 4 s at 0 force and 2-s wait time before being plunge-vitrified in liquid propane using Mark IV Vitrobot (Thermo Fisher Scientific). The blotting chamber was maintained at 4°C and 100% humidity during freezing. Movies were collected using Titan Krios (Thermo Fisher Scientific) outfitted with a K3 camera and BioQuantum energy filter (Gatan) set to 10 eV. A total of 34,712 movies were collected at a nominal magnification of ×105,000 and a physical pixel size of 0.85 Å, with a 70-μm C2 aperture and 100-μm objective aperture at a dose rate of 19.5 e^−^/pixel per second. A total dose of 81 e^−^/Å^2^ was collected with 3-s exposure as movies of 45 frames. Data were collected using EPU (Thermo Fisher Scientific).

### Cryo-EM sample preparation and data collection on *Hs*PepT1 bound to Ala-Phe in the outward-facing open state and in the outward-facing occluded state

Four microliters of purified *Hs*PepT1 at 2 mg/ml supplemented with 20 mM Ala-Phe was applied to a glow-discharged gold holey carbon 2/1 300-mesh grid (Quantifoil). The grid was blotted for 4.0 s at 0 force before being plunge-vitrified in liquid propane using Mark IV Vitrobot (Thermo Fisher Scientific). The blotting chamber was maintained at 4°C and 100% humidity during freezing. Movies were collected using Titan Krios (Thermo Fisher Scientific) equipped with a K3 camera and BioQuantum energy filter (Gatan) set to 15 eV. A total of 37,822 movies were collected in two separate sessions (16,522 in the first session and 21,300 in the second session) using EPU (Thermo Fisher Scientific) at a nominal magnification of ×130,000 and a physical pixel size of 0.67 Å, with a 70-μm C2 aperture and 100-μm objective aperture at a dose rate of 15 e^−^/pixel per second. A total dose of 55 e^−^/Å^2^ was collected with 1.7-s exposure as movies of 40 frames.

### Cryo-EM image processing of apo *Hs*PepT1 in the outward-facing open state

Movies were motion-corrected using Relion-3.1 (*62*) own implementation of MotionCor2 (*63*). Contrast transfer function parameters were calculated using CTFFIND4 (*64*). A total of 2,091,726 coordinates were extracted from 22,537 micrographs using CrYOLO (*65*), with a 200-pixel box and binning to 50 pixels, and were subjected to multiple rounds of 2D classification in Relion-3.1. A total of 1,459,348 particles were selected and reextracted with a 200-pixel box size without binning. A 3D ab initio reconstruction was generated in CryoSPARCv2 with all particles and low pass–filtered at 30 Å for 3D classification in Relion-3.1. After multiple rounds of 3D classification using *T* = 10 and *K* = 4, the best classes were selected for additional 3D classification without image alignment in Relion-3.1, focusing on the protein and excluding the micelle. The selection of 593,757 particles from 3D classes with strong signal inside the micelle and in the extracellular domain led to a reconstruction of 4.6 Å in Relion-3.1. CTF refine (per particle defocus and beam tilt) and Bayesian polishing (using optimized trained parameters on a subset of 20,000 particles) were performed in Relion-3.1. The shiny particles were then imported in CryoSPARCv3 for nonuniform refinement (*66*) that led to a 3.9-Å reconstruction estimated in cryoSPARCv3 using the Fourier shell correlation (FSC) = 0.143 cutoff. Local resolution estimations were calculated in CryoSPARCv3 using 0.5 FSC cutoff. Postprocessing in DeepEMhancer (*67*) using the two half maps as input and the default tightTarget model resulted in a more interpretable map, which was used only for illustration purposes in Fig. 1B, while the model was build and refined in a map postprocessed using default parameters in the Phenix Autosharpen utility (*68*).

### Cryo-EM image processing of *Hs*PepT2 bound to Ala-Phe in the inward-facing partially occluded state

Movies were motion-corrected using Relion-3.1 (*62*) own implementation of MotionCor2 (*63*). Contrast transfer function parameters were calculated using CTFFIND4 (*64*). A total of 4,388,314 coordinates were extracted from 34,712 micrographs using CrYOLO (*65*), with a 200-pixel box and binning to 50 pixels, and were subjected to multiple rounds of 2D classification in Relion-3.1. A total of 2,944,737 particles were selected and reextracted with a 200-pixel box size without binning. A 3D ab initio reconstruction was generated in CryoSPARCv2 with a subset of particles and low pass–filtered at 30 Å for 3D refinement in Relion-3.1 on the 2,944,737 particles, yielding a 7.5-Å reconstruction. After multiple rounds of 3D classification without image alignment in Relion-3.1, using *T* = 4, 8, 10, 20, 30, and 40 focusing on the protein and excluding the micelle, the selection of 454,149 particles from 3D classes with strong signal inside the micelle and in the extracellular domain led to a reconstruction of 4.3 Å in Relion-3.1 using SIDESPLITTER (*69*) and a soft mask covering the micelle and the extracellular domain. Bayesian polishing was performed in Relion-3.1 using optimized trained parameters on a subset of 20,000 particles. The shiny particles were then imported in CryoSPARCv3 for CTF Refinement per particle (defocus and beamtilt). Nonuniform refinement (*66*) led to a 3.8-Å reconstruction estimated in cryoSPARCv3 using the FSC = 0.143 cutoff. Local resolution estimations were calculated in CryoSPARCv3 using 0.5 FSC cutoff. Postprocessing in DeepEMhancer (*67*) using the two half maps as input and the default tightTarget model resulted in a more interpretable map, which was used only for illustration purposes in Fig. 1C, while the model was build and refined in a map postprocessed using default parameters in the Phenix Autosharpen utility (*68*).

### Cryo-EM image processing of *Hs*PepT1 bound to Ala-Phe in the outward-facing open and occluded states

Movies were motion-corrected using Relion-3.1 (*62*) own implementation of MotionCor2 (*63*). Contrast transfer function parameters were calculated using CTFFIND4 (*64*). A total of 6,046,602 coordinates were extracted from 37,822 micrographs using CrYOLO (*65*), with a 200-pixel box and binning to 50 pixels, and were subjected to multiple rounds of 2D classification in Relion-3.1. A total of 4,247,238 particles were selected and reextracted with a 200-pixel box size without binning. The 3D volume of apo *Hs*PepT1 in its outward-facing open conformation was low pass–filtered at 40 Å for 3D classification in Relion-3.1, resulting in a reconstruction at 4.1 Å with 486,562 particles for the first dataset, and 4.5 Å with 599,754 particles for the second, after performing Bayesian polishing using individually optimized trained parameters on a subset of 30,000 particles for each dataset. In both datasets, further 3D classifications allowed to separate two distinct conformational states, including the substrate-bound outward-facing open state and the substrate-bound outward-facing occluded state differing by the opening or closure of TM2. Focused 3D classification without alignment on the transmembrane domain in Relion-3.1 allowed to improve the resolution of the substrate-bound outward-facing open state from 3.7 to 3.5 Å after nonuniform refinement (*66*) in cryoSPARCv3. The substrate-bound outward-facing occluded state reached a modest resolution of 4.1 Å after nonuniform refinement in cryoSPARCv3 but led to a clear resolution of all TMs, including the closed TM2. For the 3.5-Å resolution substrate-bound outward-facing open reconstruction, postprocessing was done in Phenix using default parameters in the Autosharpen utility (*68*). This map was subsequently used for model building and refinement. For illustration purposes in Fig. 5E only, the half maps were also subjected to postprocessing in DeepEMhancer (*67*) using the two half maps as input and the default tightTarget model. For the 4.1-Å resolution substrate-bound outward-facing occluded reconstruction, postprocessing was done in cryoSPARCv3.

### Model building and refinement

The transmembrane domain of *Hs*PepT2 was built manually in Coot (*70*), guided by secondary structure predictions from PSIPRED (*71*). The resolution in the extracellular domain of *Hs*PepT2 did not allow de novo model building. Instead, the structure of *Hs*PepT2-ECD predicted by AlphaFold (*50*) was docked in the cryo-EM density and linked to the transmembrane domain. The model was iteratively adjusted and refined using Isolde (*72*) and Phenix real-space refine (*73*). An inward open partially occluded model of *Hs*PepT1 was generated in SWISSMODEL (*74*), using *Hs*PepT2 as a reference template. The model was then subjected to multiple rounds of refinement in NAMDINATOR (*75*), Coot, and Phenix real-space refine (*73*) to fit in the apo outward-facing open reconstruction. Once the model fitted the density, the extracellular domain was replaced by the AlphaFold *Hs*PepT1-ECD prediction, which improved the quality of the model significantly. This model was then subjected to adjustments in Isolde and a final refinement using Phenix real-space refine. The substrate-bound outward-facing open and occluded models were generated using the final apo outward-facing open model, and manual adjustments in Coot and Isolde before real-space refinement in Phenix. Validation of the models was performed using MolProbity in Phenix.

## Notes

View/request a protocol for this paper from *Bio-protocol*.
