# Supplementary material for: Structural snapshots of human PepT1 and PepT2 reveal mechanistic insights into substrate and drug transport across epithelial membranes
Source: Sci Adv. 2021 Nov 3;(7):eabk3259. doi: 10.1126/sciadv.abk3259 (PMC8565842; doi:10.1126/sciadv.abk3259)
Supplement: Supplementary file 1 — Figs. S1 to S16 Table S1 Legends for movie S1 [file sciadv.abk3259_sm.pdf]

Science Advances
AAAS

Supplementary Materials for

Structural snapshots of human PepT1 and PepT2 reveal mechanistic insights into substrate and drug transport across epithelial membranes

Maxime Killer, Jiri Wald, Joanna Pieprzyk, Thomas C. Marlovits, Christian Löw*

*Corresponding author. Email: christian.loew@embl-hamburg.de

Published 3 November 2021, *Sci. Adv.* **7**, eabk3259 (2021)
DOI: 10.1126/sciadv.abk3259

The PDF file includes:

Figs. S1 to S16
Table S1
Legends for movie S1

Other Supplementary Material for this manuscript includes the following:

Movie S1

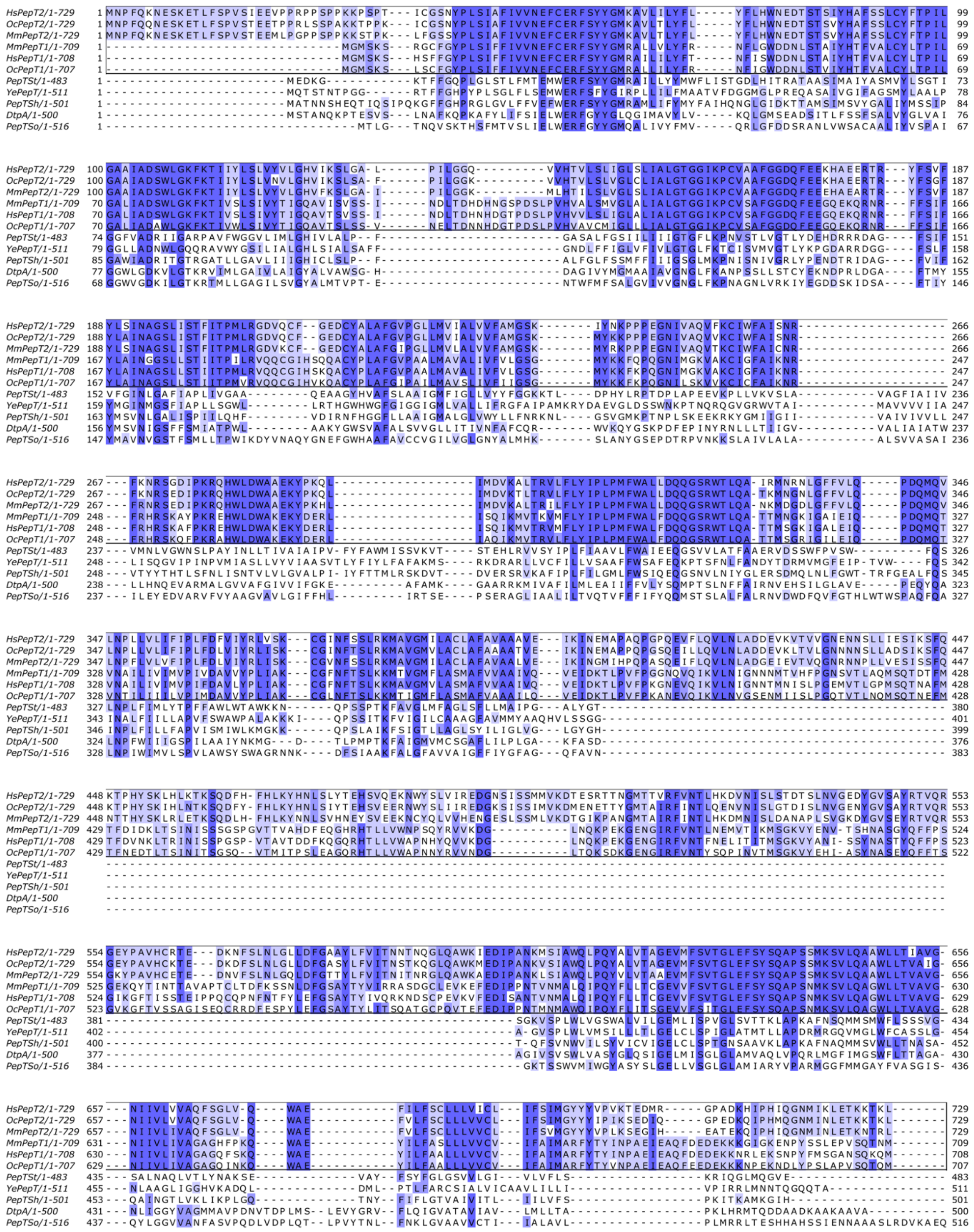

Fig. S1. Multiple sequence alignment of mammalian and bacterial POTs colored by sequence conservation. Mammalian POTs from top to bottom: *Hs*PepT2 (Q16348), *Oc*PepT2 (P46029), *Mm*PepT2 (Q9ES07), *Mm*PepT1 (Q9JIP7), *Hs*PepT1 (P46059), *Oc*PepT1 (P36836). Bacterial POTs from top to bottom: PepT$_{St}$ (Q5M4H8), *Ye*PepT (A0A2R9TD79), PepT$_{Sh}$ (A0A657M1C3), DtpA (P77304), PepT$_{So}$ (Q8EHE6). Number in parenthesis correspond to UniProt entries.

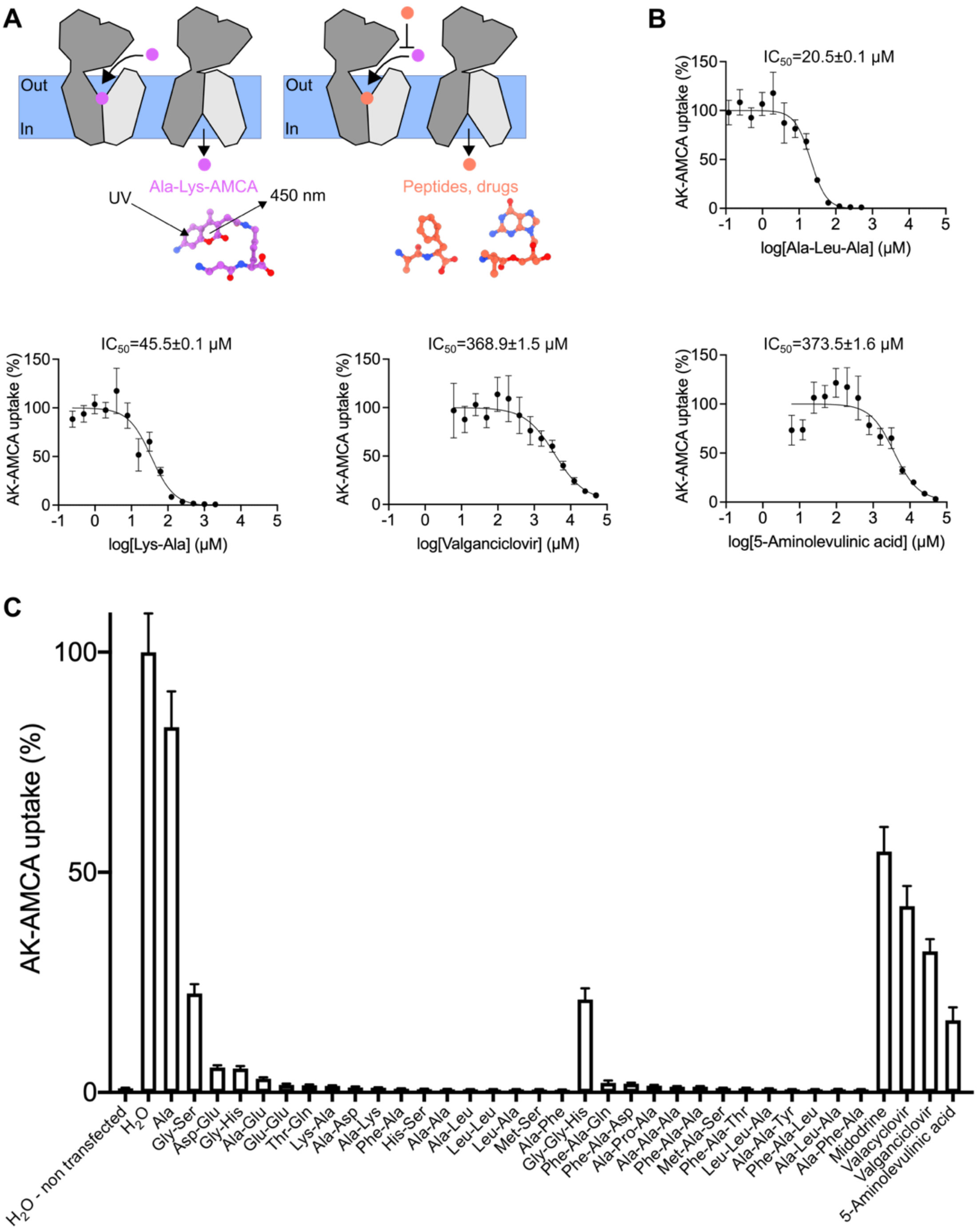

Fig. S2. Whole cell transport assays of the β-Ala-Lys peptide coupled to the fluorescent reporter AMCA (AK-AMCA) in *Hs*PepT2 transfected HEK293F cells in absence or presence of dipeptides, tripeptides, or drugs as schematized in (A). (B) Concentration dependent competition of the fluorescent reporter with the stated peptide or drug. **(C)** The assay was repeated on several substrates competing at a concentration of 5 mM. The average uptake value for each condition was calculated from three independent measurements. The error bars correspond to the standard deviation from these independent measurements.

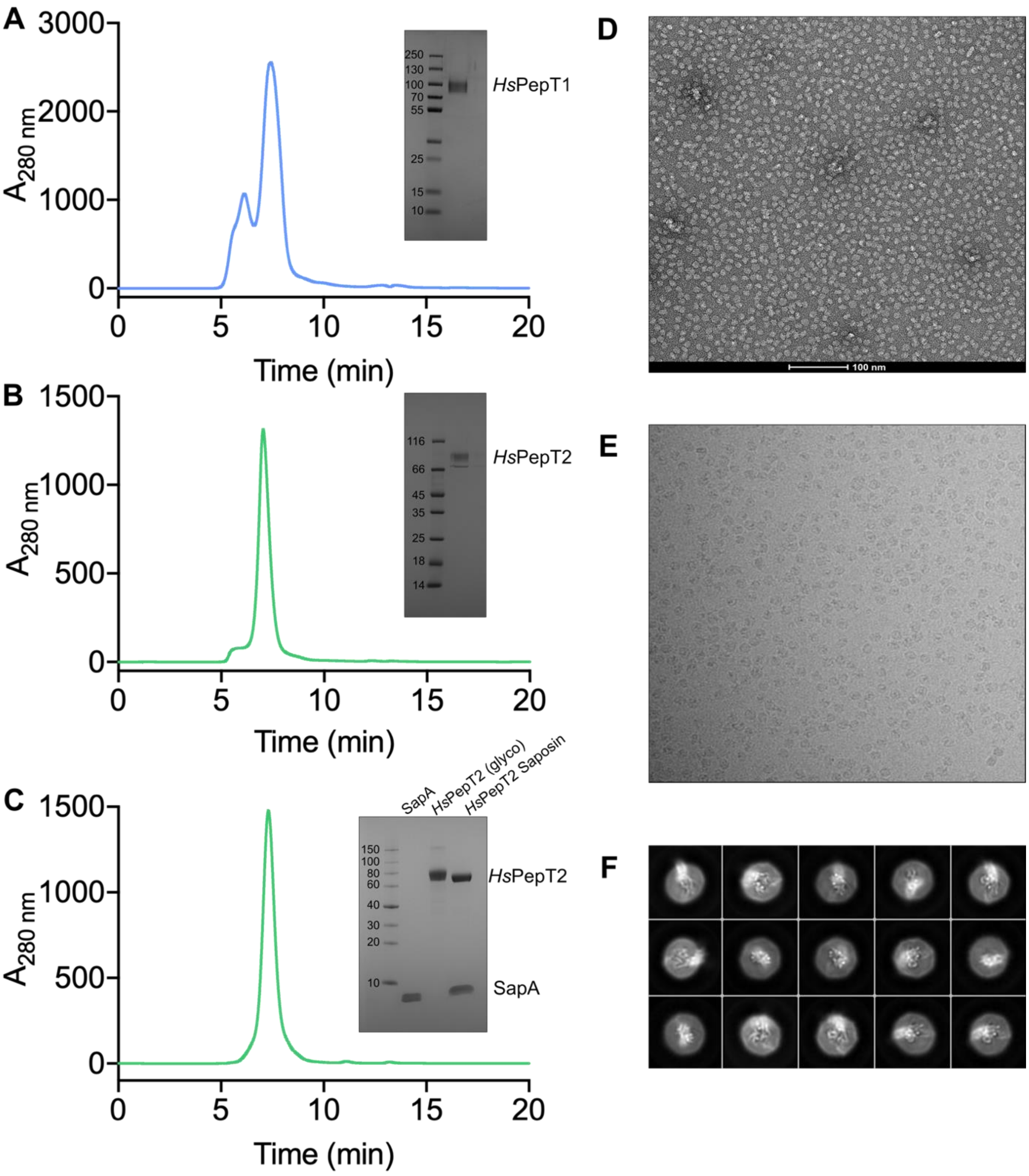

Fig. S3. Purifications of *Hs*PepT1 and *Hs*PepT2. SEC chromatograms and corresponding SDS PAGE of purified (A) *Hs*PepT1 in detergent (B) *Hs*PepT2 in detergent (C) *Hs*PepT2 reconstituted in Saposin-brain lipid nanoparticles. (D, E, F) Negative stain, cryo-EM micrograph, and 2D class averages of *Hs*PepT2 reconstituted in Saposin-brain lipid nanoparticles.

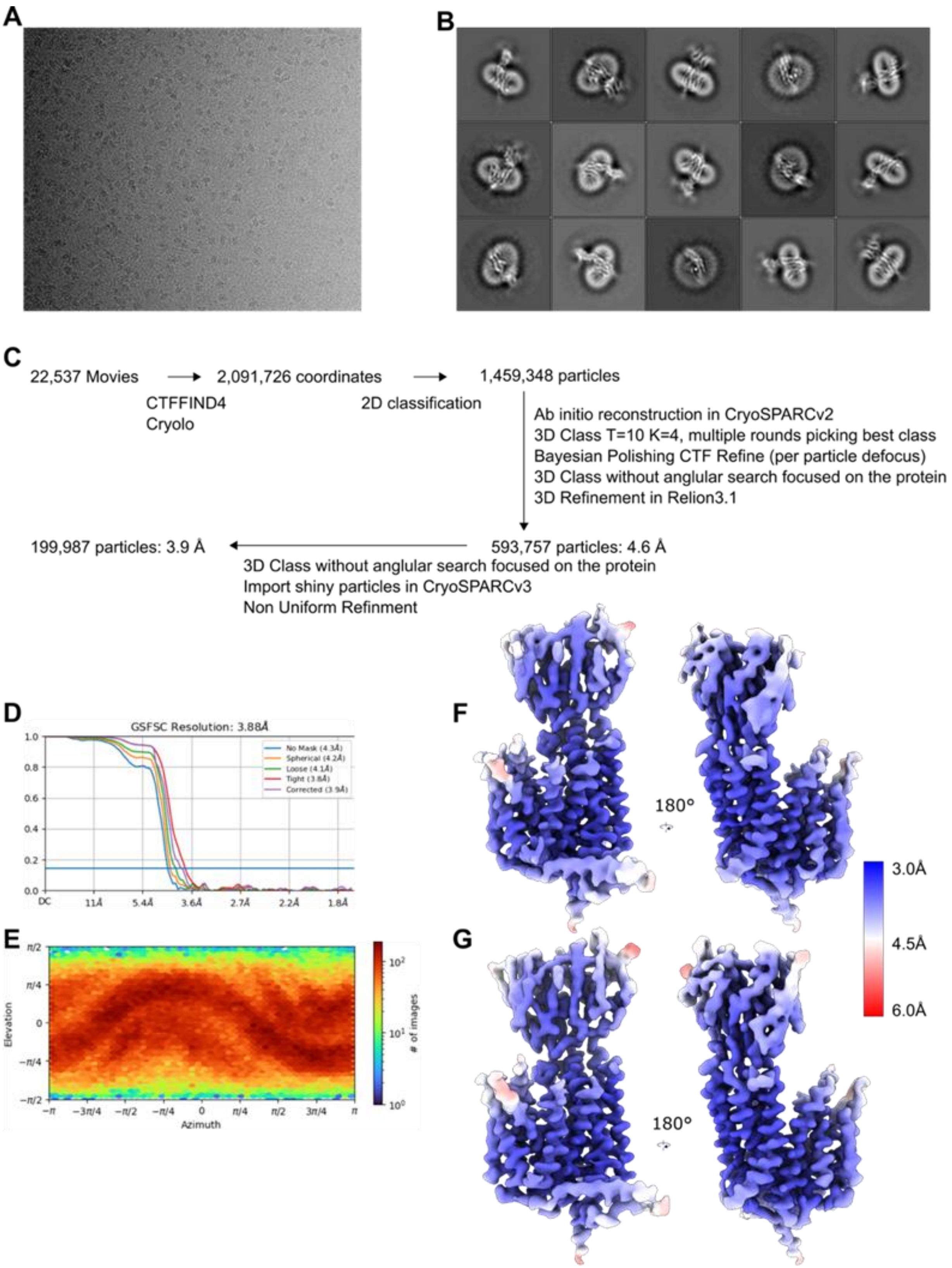
A
B
C
22,537 Movies
CTFFIND4
Cryolo
2,091,726 coordinates
2D classification
1,459,348 particles
Ab initio reconstruction in CryoSPARCv2
3D Class T=10 K=4, multiple rounds picking best class
Bayesian Polishing CTF Refine (per particle defocus)
3D Class without angular search focused on the protein
3D Refinement in Relion3.1
593,757 particles: 4.6 Å
3D Class without anglular search focused on the protein
Import shiny particles in CryoSPARCv3
Non Uniform Refinment
199,987 particles: 3.9 Å
D
GSFSC Resolution: 3.88Å
No Mask (4.3Å)
Spherical (4.2Å)
Loose (4.1Å)
Tight (3.8Å)
Corrected (3.9Å)
DC
11Å
5.4Å
3.6Å
2.7Å
2.2Å
1.8Å
E
Elevation
Azimuth
# of images
F
G
180°
3.0Å
4.5Å
6.0Å

Fig. S4. Cryo-EM data processing for outward facing open apo *Hs*PepT1. (A) Representative motion-corrected micrograph collected on the Titan Krios. (B) Examples of 'good' 2D class averages that were used in 3D classification. (C) Flowchart showing the image processing pipeline. Initial processing was performed in Relion-3.1. Particles were then transferred to cryoSPARCv3 for Non Uniform refinement. The numbers of particles moving into each step are noted. (D) Final refinement from cryoSPARCv3 FSC curve. (E) angular distribution. (F) Phenix auto sharpened postprocessed map used for model building and real space refinement, colored by local resolution, estimated in cryoSPARCv3 using the 0.5 as FSC threshold. (G) deepEMhancer postprocessed map used only for illustration in Fig. 1B, colored by local resolution, estimated in cryoSPARCv3 using the 0.5 as FSC threshold.

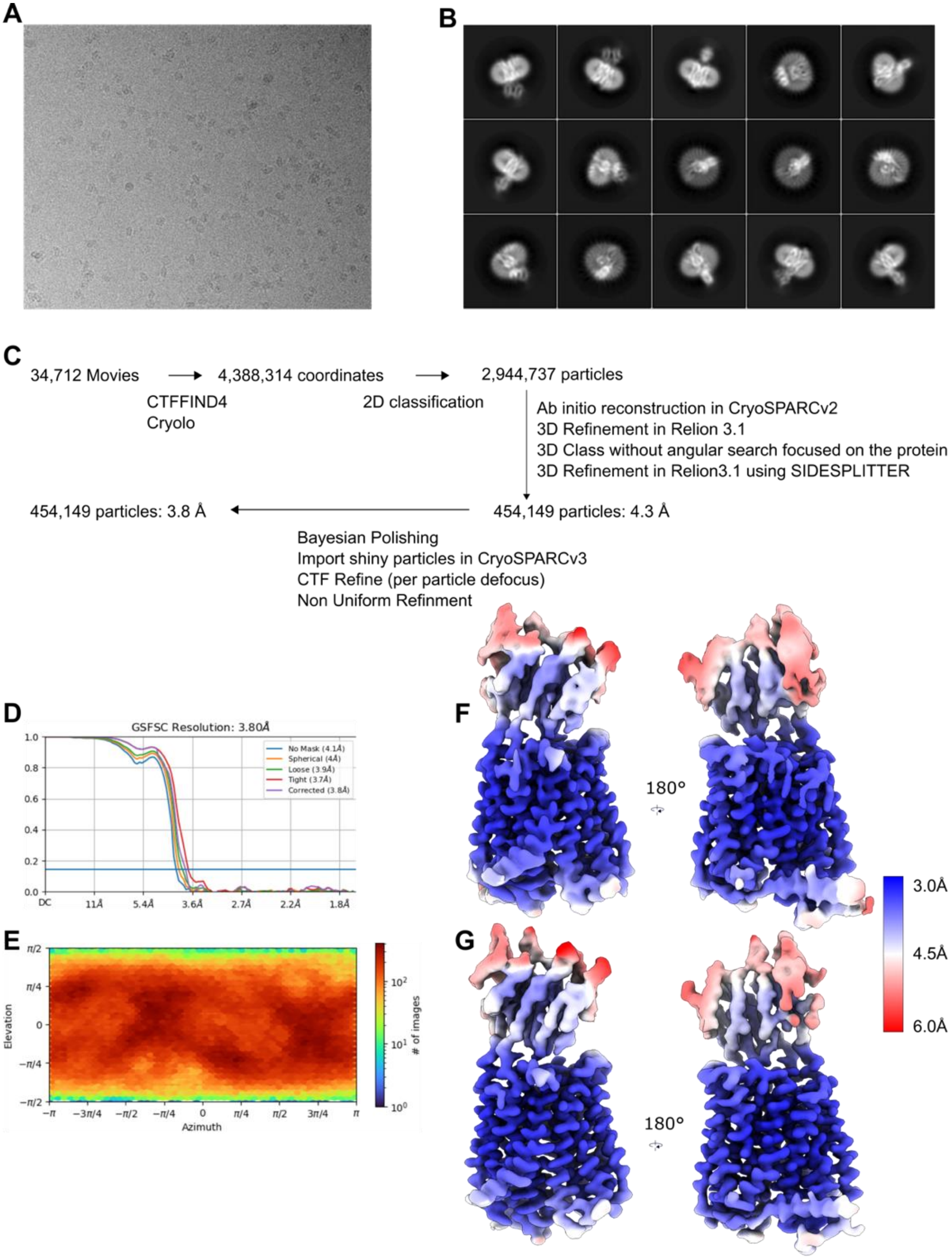
A
B
C
34,712 Movies
4,388,314 coordinates
2,944,737 particles
CTFFIND4
Cryolo
2D classification
Ab initio reconstruction in CryoSPARCv2
3D Refinement in Relion 3.1
3D Class without angular search focused on the protein
3D Refinement in Relion3.1 using SIDESPLITTER
454,149 particles: 3.8 Å
454,149 particles: 4.3 Å
Bayesian Polishing
Import shiny particles in CryoSPARCv3
CTF Refine (per particle defocus)
Non Uniform Refinment
D
GSFSC Resolution: 3.80Å
No Mask (4.1Å)
Spherical (4Å)
Loose (3.9Å)
Tight (3.7Å)
Corrected (3.8Å)
DC
11Å
5.4Å
3.6Å
2.7Å
2.2Å
1.8Å
E
Elevation
Azimuth
# of images
F
G
180°
180°
3.0Å
4.5Å
6.0Å

Fig. S5. Cryo-EM data processing for inward facing partially occluded *Hs*PepT2 bound to Ala-Phe. (A) Representative motion-corrected micrograph collected on the Titan Krios. (B) Examples of ‘good’ 2D class averages that were used in 3D classification. (C) Flowchart showing the image processing pipeline. Initial processing was performed in Relion-3.1. Particles were then transferred to cryoSPARCv3 for CTF-Refinement and Non Uniform refinement. The numbers of particles moving into each step are noted. (D) Final refinement from cryoSPARCv3 FSC curve. (E) angular distribution. (F) Phenix auto sharpened postprocessed map used for model building and real space refinement, colored by local resolution, estimated in cryoSPARCv3 using the 0.5 as FSC threshold. (G) deepEMhancer postprocessed map used only for illustration in Fig. 1C, colored by local resolution, estimated in cryoSPARCv3 using the 0.5 as FSC threshold.

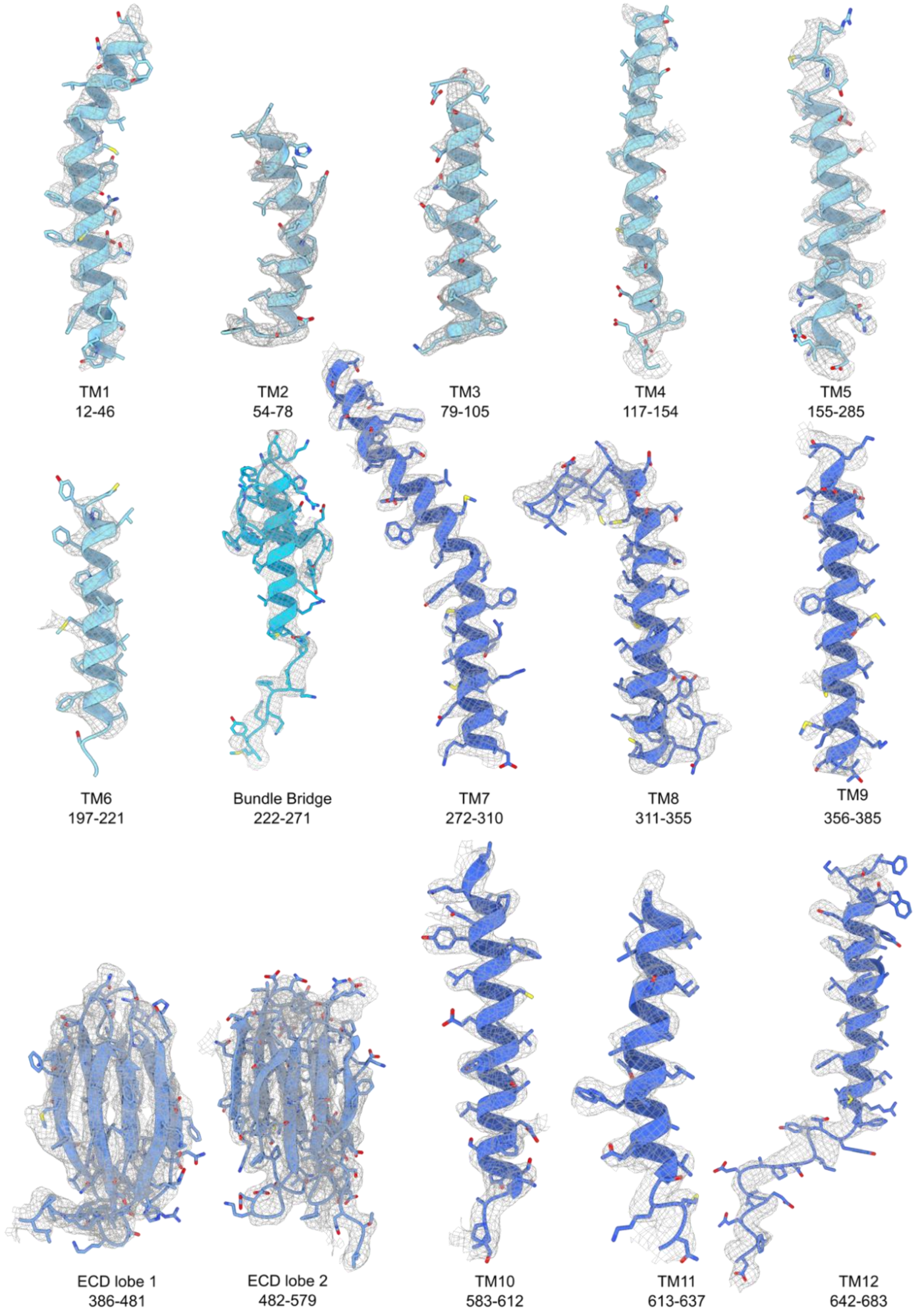

Fig. S6. Cryo-EM map density of outward facing open apo *Hs*PepT1. The density is shown as grey mesh for individual transmembrane helices, bundle bridge and the extracellular domain. The mesh depicts density within a 2.6 Å radius of any modelled atom.

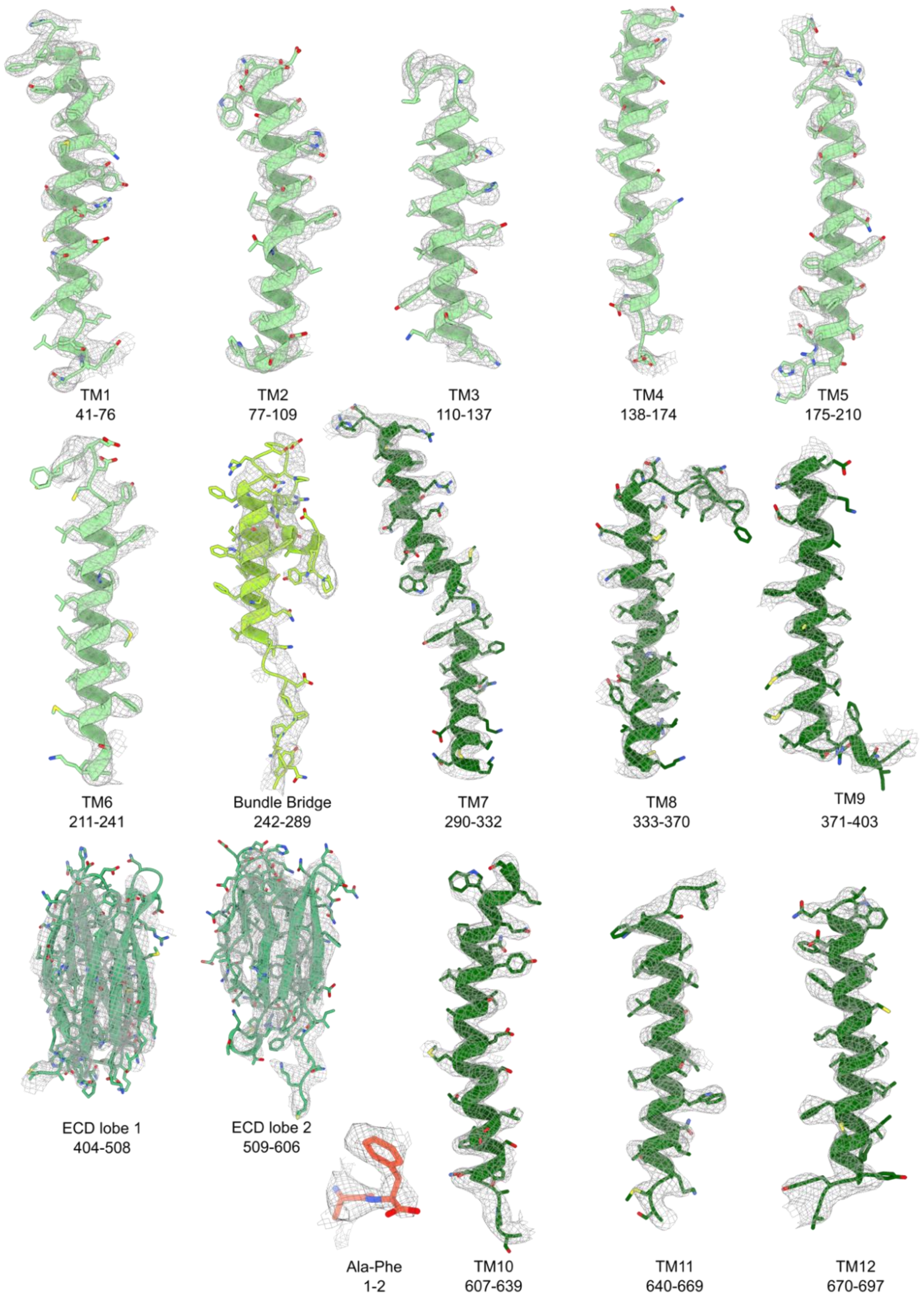

Fig. S7. Cryo-EM map density of inward facing partially occluded *Hs*PepT2 bound to Ala-Phe. The density is shown as grey mesh for individual transmembrane helices, bundle bridge and the extracellular domain. The mesh depicts density within a 2.6 Å radius of any modelled atom.

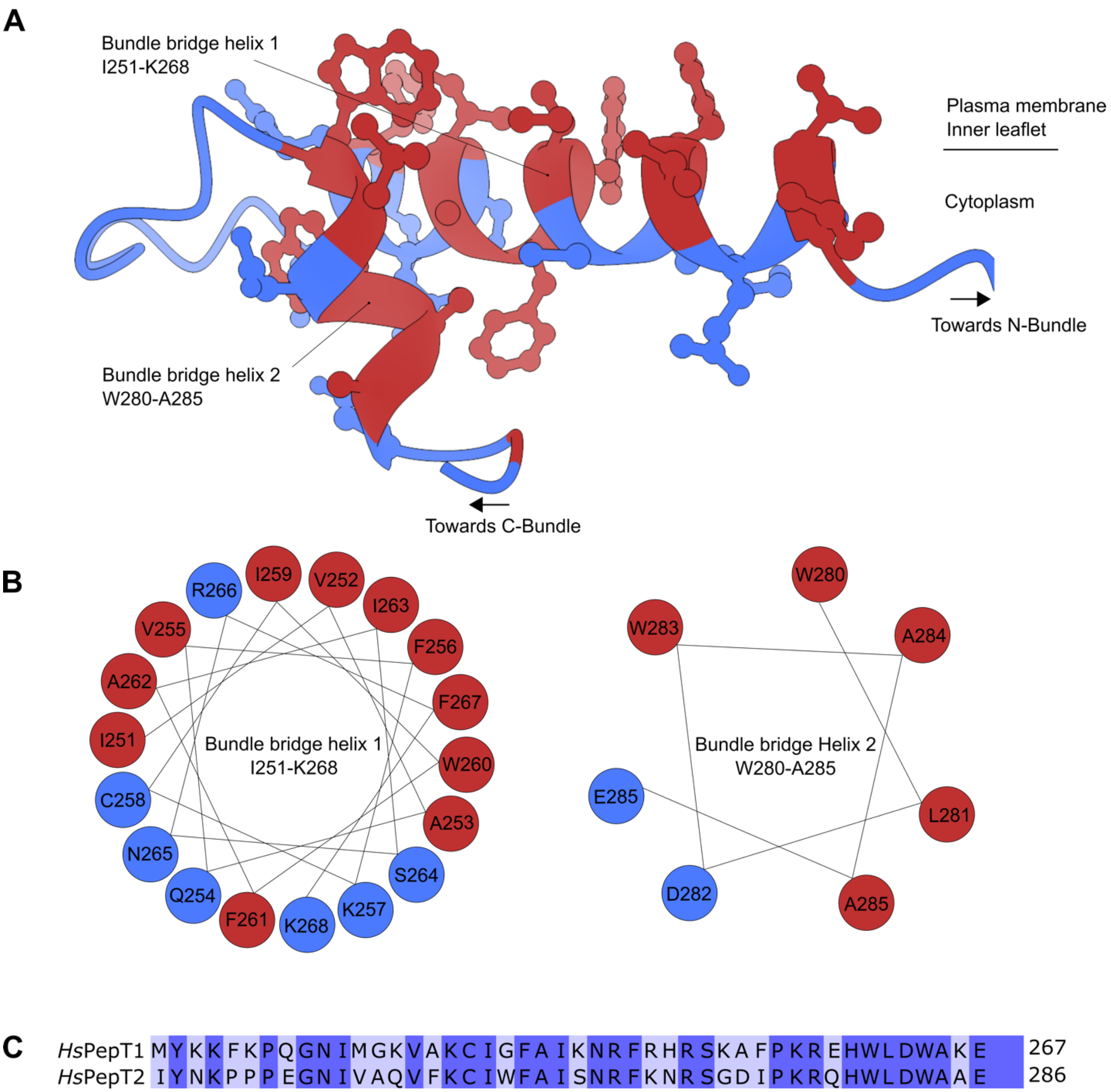

Fig. S8. Amphipathic nature of the bundle bridge. (A) Ribbon representation of *Hs*PepT2 bundle bridge. Polar residues are colored in blue, hydrophobic residues are colored in red. (B) Edmundson wheel projection diagram of the bundle bridge helices 1 and 2 showing the concentration of hydrophobic residues facing the inner leaflet of the plasma membrane and the presence of polar residues facing the cytoplasm. (C) Sequence alignment of the bundle bridge from *Hs*PepT1 and *Hs*PepT2.

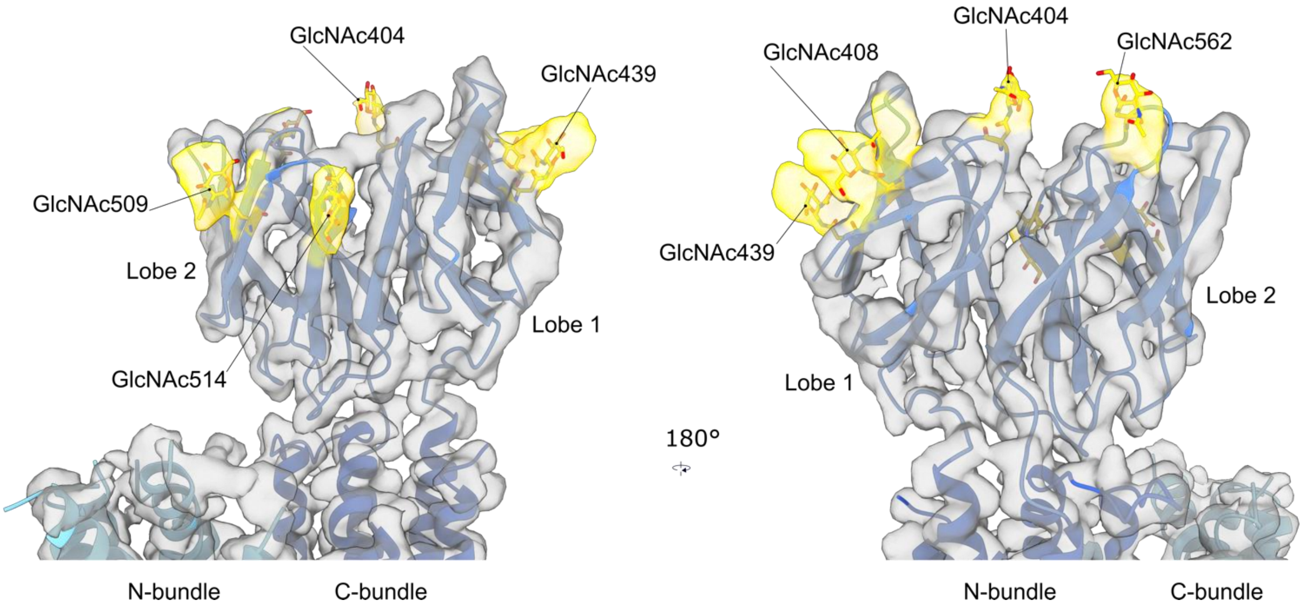

Fig. S9. N-Glycans present on the extracellular domain of *Hs*PepT1. Residues N404, N408, N439, N509, N514 and N562 are glycosylated.

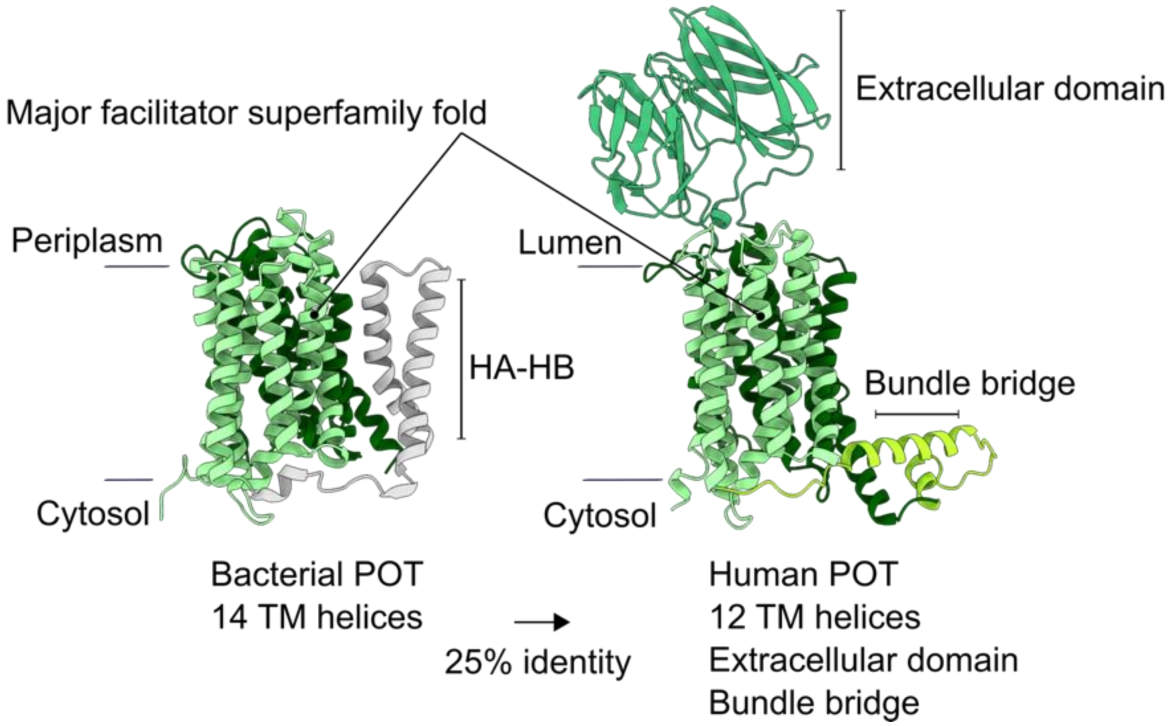

Fig. S10. Architectural differences between bacterial and human POTs. While bacterial POTs are composed of 14 transmembrane helices, human homologues contain a transporter unit of 12 transmembrane helices, an extracellular domain and the connecting bundle bridge.

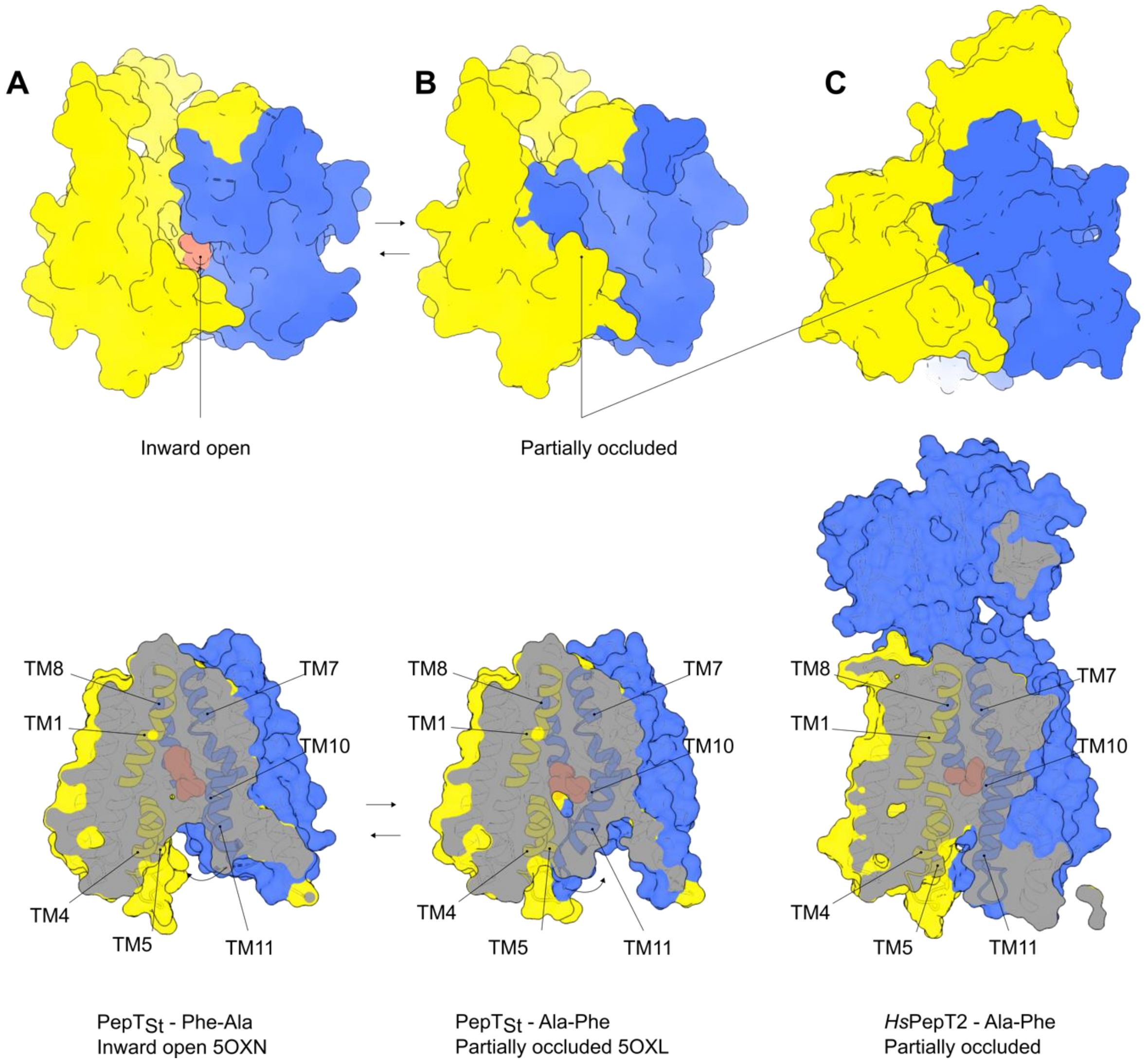

Fig. S11. Occlusion of the cytosolic side in bacterial POTs occurs *via* bending of the TM10/TM11 hairpin. (A) PepT$_{St}$ inward open structure is characterized by a tight sealing on the periplasmic side mediated by TM1, TM7 and TM8 while the substrate is accessible to the solvent on the cytoplasmic side as illustrated in the surface representation and the cutaway surface side view. (B) TM10 and TM11 can come closer to TM4 and TM5 to partially occlude the solvent access from the cytoplasmic side (C) In *Hs*PepT2, the extracellular space is sealed in a similar fashion, and the cytoplasmic side is partially closed, representing a state between fully open and occluded.

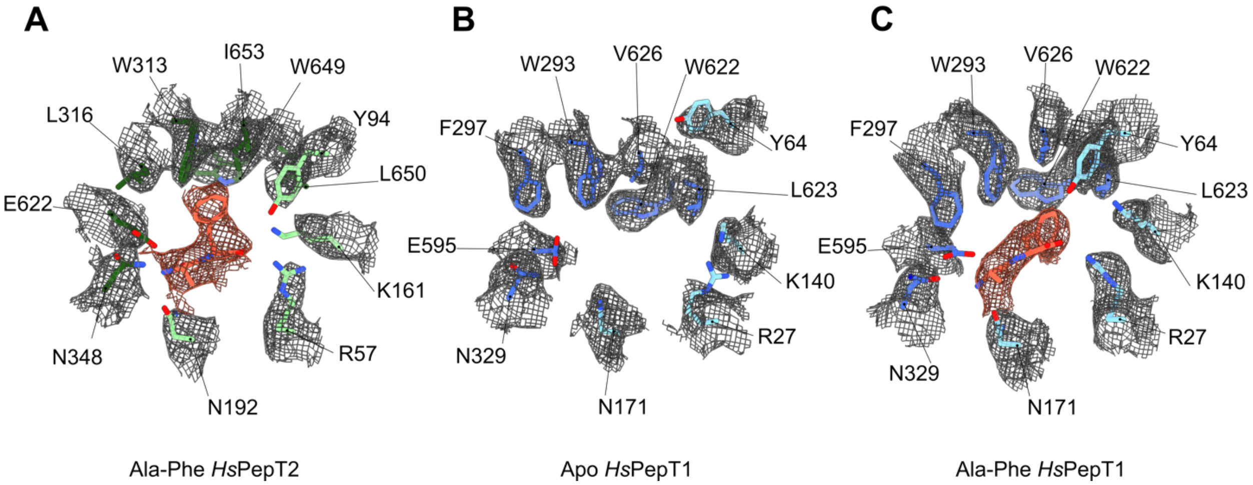

Fig. S12. Cryo-EM density map of the substrate binding site of (A) inward facing partially occluded *Hs*PepT2 bound to Ala-Phe (B) outward facing open apo *Hs*PepT1 and (C) outward facing open *Hs*PepT1 bound to Ala-Phe.

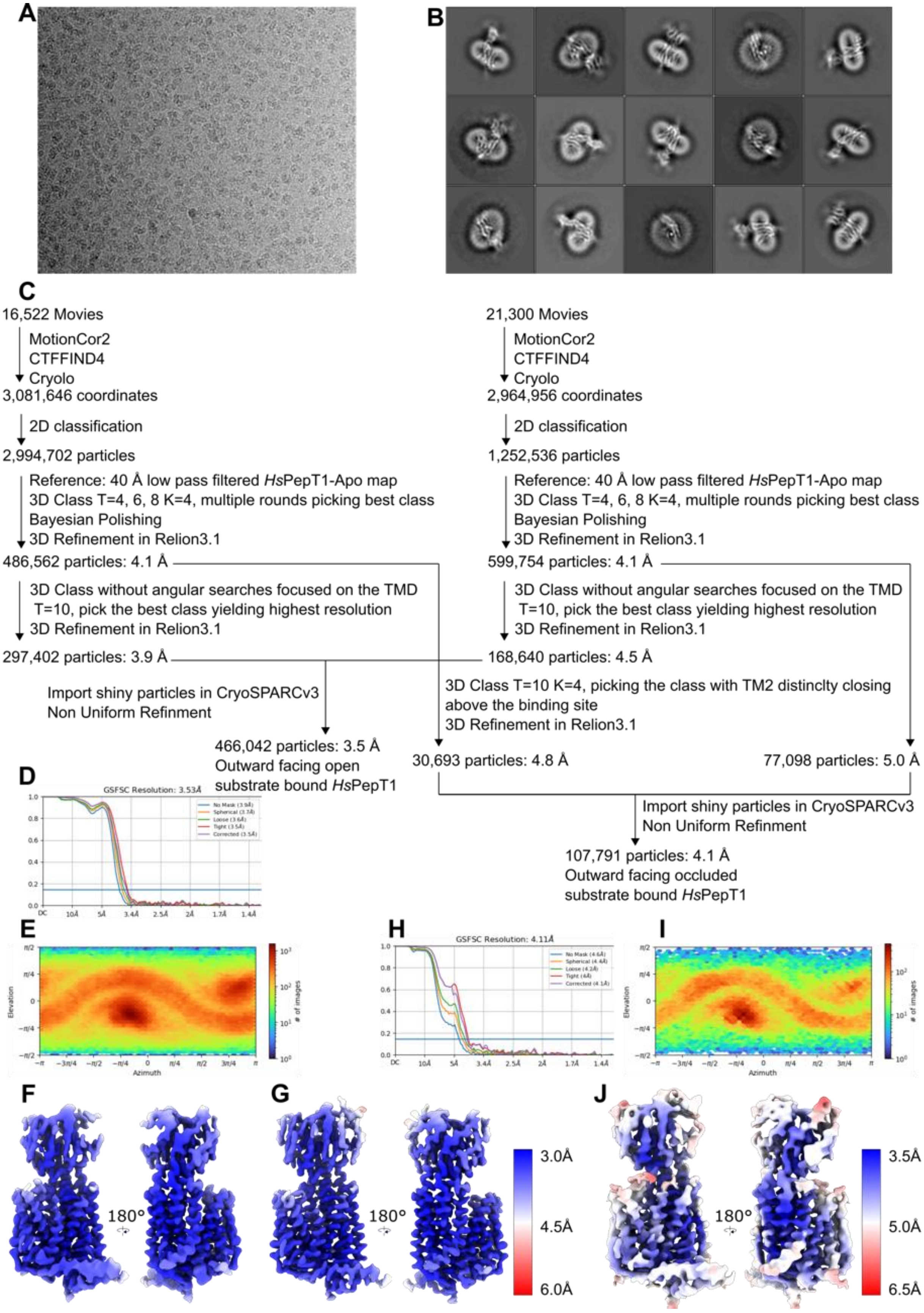
A
B
C
16,522 Movies
MotionCor2
CTFFIND4
Cryolo
3,081,646 coordinates
2D classification
2,994,702 particles
Reference: 40 Å low pass filtered HsPepT1-Apo map
3D Class T=4, 6, 8 K=4, multiple rounds picking best class
Bayesian Polishing
3D Refinement in Relion3.1
486,562 particles: 4.1 Å
3D Class without angular searches focused on the TMD
T=10, pick the best class yielding highest resolution
3D Refinement in Relion3.1
297,402 particles: 3.9 Å
Import shiny particles in CryoSPARCv3
Non Uniform Refinment
466,042 particles: 3.5 Å
Outward facing open
substrate bound HsPepT1
21,300 Movies
MotionCor2
CTFFIND4
Cryolo
2,964,956 coordinates
2D classification
1,252,536 particles
Reference: 40 Å low pass filtered HsPepT1-Apo map
3D Class T=4, 6, 8 K=4, multiple rounds picking best class
Bayesian Polishing
3D Refinement in Relion3.1
599,754 particles: 4.1 Å
3D Class without angular searches focused on the TMD
T=10, pick the best class yielding highest resolution
3D Refinement in Relion3.1
168,640 particles: 4.5 Å
3D Class T=10 K=4, picking the class with TM2 distinclty closing
above the binding site
3D Refinement in Relion3.1
30,693 particles: 4.8 Å
77,098 particles: 5.0 Å
Import shiny particles in CryoSPARCv3
Non Uniform Refinment
107,791 particles: 4.1 Å
Outward facing occluded
substrate bound HsPepT1
D
GSFSC Resolution: 3.53Å
E
H
GSFSC Resolution: 4.11Å
I
Azimuth
Elevation
# of images
F
G
J
180°
3.0Å
4.5Å
6.0Å
3.5Å
5.0Å
6.5Å

Fig. S13. Cryo-EM data processing for outward facing open and outward facing occluded *Hs*PepT1 bound to Ala-Phe. (A) Representative motion-corrected micrograph collected on the Titan Krios. (B) Examples of 'good' 2D class averages that were used in 3D classification. (C) Flowchart showing the image processing pipeline. Initial processing was performed in Relion-3.1. Particles were then transferred to cryoSPARCv3 for CTF-Refinement and Non Uniform refinement. The numbers of particles moving into each step are noted. (D, E, F, G) Final refinement of the outward facing open substrate bound state from cryoSPARCv3. (D) FSC curve. (E) angular distribution. (F) Phenix auto sharpened postprocessed map used for model building and real space refinement, colored by local resolution, estimated in cryoSPARCv3 using the 0.5 as FSC threshold. (G) deepEMhancer postprocessed map used only for illustration in Fig. 5E, colored by local resolution, estimated in cryoSPARCv3 using the 0.5 as FSC threshold. (H, I, J) Final refinement of the outward facing occluded substrate bound state from cryoSPARCv3. (H) FSC curve. (I) angular distribution. (J) Phenix auto sharpened postprocessed map used for model building and real space refinement, colored by local resolution, estimated in cryoSPARCv3 using the 0.5 as FSC threshold.

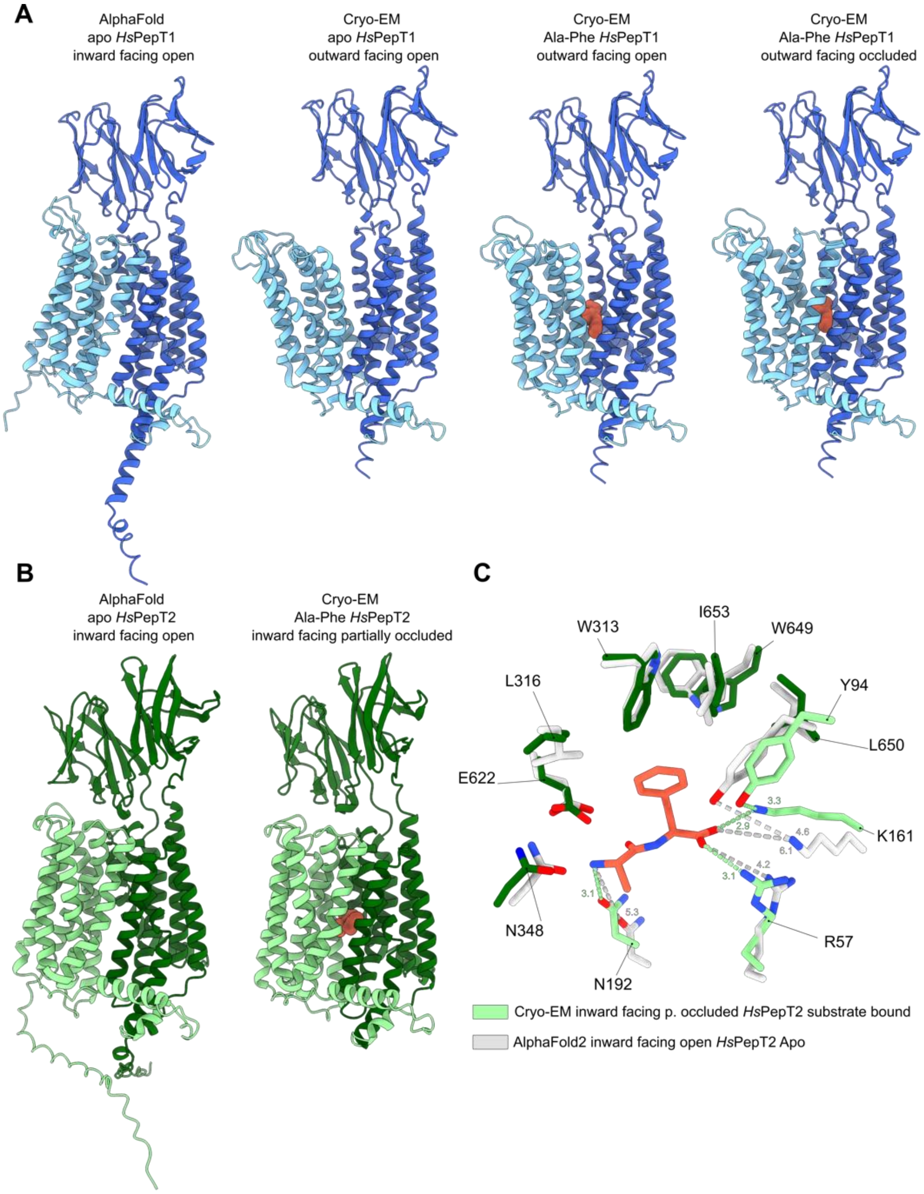

Fig. S14. Comparison of the experimental structures and predicted structures from AlphaFold. (A) Side by side comparison of the different states obtained for *Hs*PepT1 with the AlphaFold prediction. (B) Side by side comparison of the state obtained for *Hs*PepT2 with the AlphaFold prediction. (C) Overlay of the binding site between the Ala-Phe bound inward facing partially occluded *Hs*PepT2 and the predicted apo inward facing open structure from AlphaFold. Distance measurements between the dipeptide and important residues of the inward facing partially occluded Ala-Phe bound *Hs*PepT2 are indicated by dashes and numbers in Ångstroms. Crucial interactions between the transporter and the peptide termini are lost in the inward facing open apo state predicted by AlphaFold, as indicated by the distance measurements in grey.

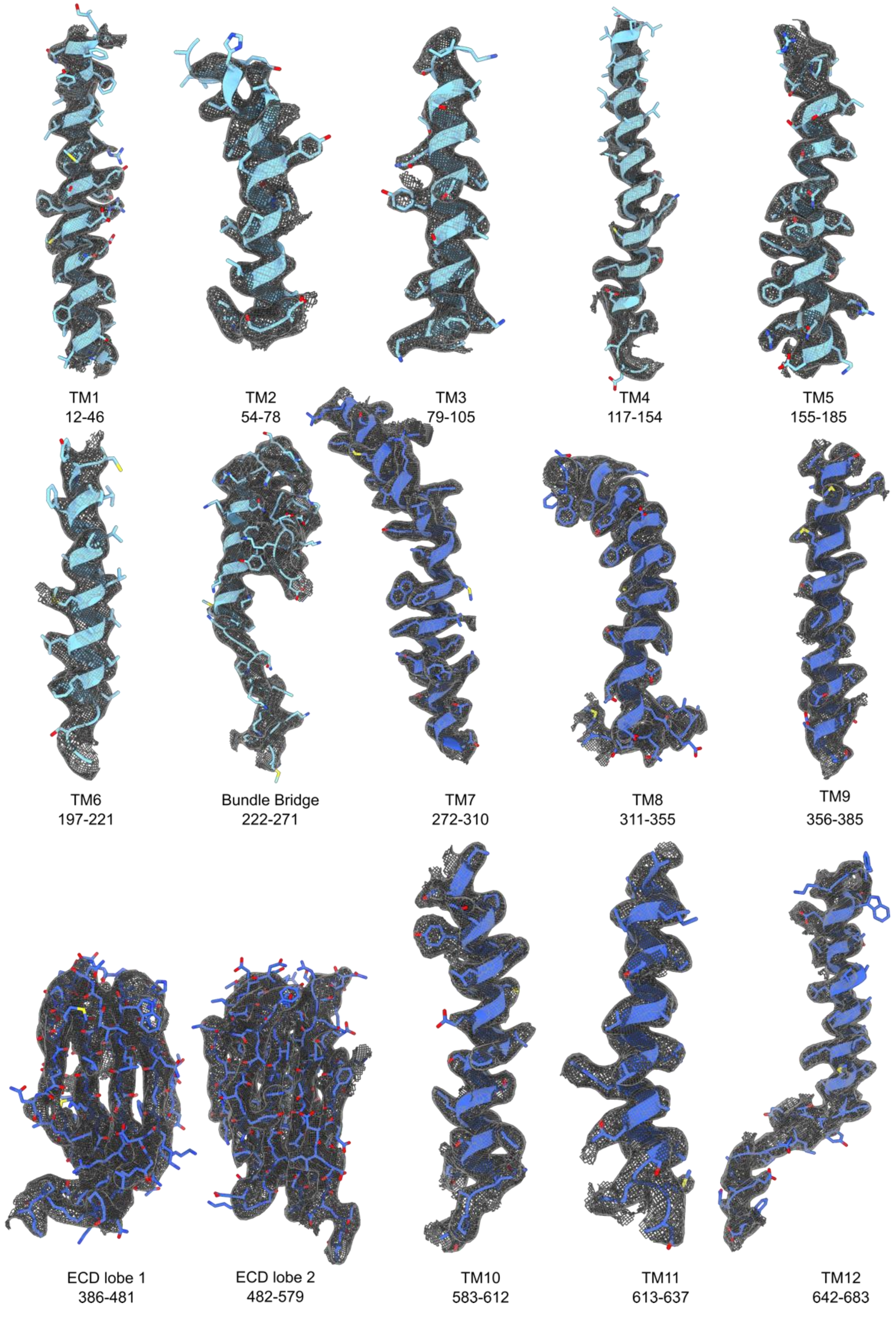

Fig. 15. Cryo-EM map density of outward facing open *Hs*PepT1 bound to Ala-Phe. The density is shown as grey mesh for individual transmembrane helices, bundle bridge and the extracellular domain. The mesh depicts density within a 2.6 Å radius of any modelled atom.

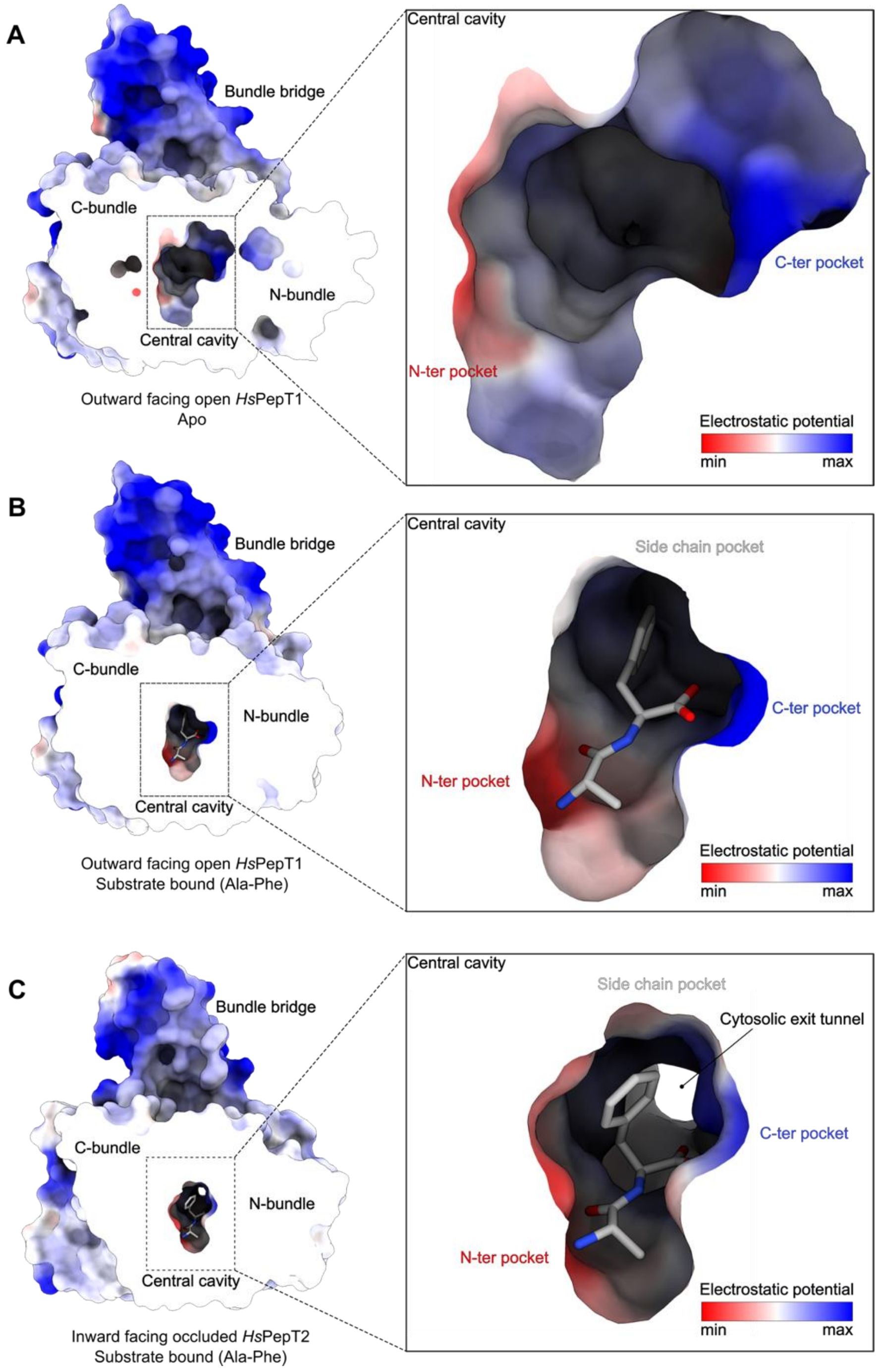

Fig. S16. Development of the central cavity upon substrate binding and its transition to the inward facing state colored by the electrostatic potential. (A) In the substrate free, outward facing conformation the central cavity has a dipole character and is widely open to the outside and can accommodate substrate of various chemical composition and molecular weights. (B) Upon binding of Ala-Phe, the N-bundle rearrangements lead to a tightening of the central cavity to fit the peptide and clamp it by its charged termini. (C) Upon switching to the inward facing state, the bi-polarity of the cavity is maintained to keep the substrate bound prior release to the cytoplasm.

Table S1. Data collection and refinement statistics of deposited structures

**Data collection**	*Hs*PepT2, Ala-Phe Inward facing partially occluded	*Hs*PepT1, Apo Outward facing open	*Hs*PepT1 Ala-Phe Outward facing open	*Hs*PepT1 Ala-Phe Outward facing occluded
Microscope/Detector	Titan Krios/Gatan K3	Titan Krios/Gatan K3	Titan Krios/Gatan K3	Titan Krios/Gatan K3
Imaging software	EPU	EPU	EPU	EPU
Magnification	105,000	105,000	130,000	130,000
Voltage (kV)	300	300	300	300
Electron exposure (e-/Å^2)	81	66	55	55
Dose rate (e-/pix/s)	19.5	16	15	15
Frame exposure (e-/Å^2)	1.8	1.3	1.4	1.4
Defocus range (µm)	-1.2 to -2.5	-0.75 to -2.5	-1.0 to -2.0	-1.0 to -2.0
Pixel size (Å)	0.85 (physical)	0.85 (physical)	0.67 (physical)	0.67 (physical)
Micrographs	34,712	22,537	37,822	37,822
**Reconstruction**				
Picked coordinates (cryolo)	4,388,314	2,091,726	6,046,602	6,046,602
Particles in 3D classification (RELION)	2,944,737	1,459,348	4,247,238	4,247,238
Particles in final refinement (CryoSPARC)	454,149	199,987	466,042	107,791
Symmetry imposed	C1	C1	C1	C1
Map sharpening method /Final map kurtosis	Phenix Autosharpen / 130.45	Phenix Autosharpen / 75.88	Phenix Autosharpen / 52.35	Phenix Autosharpen / 54.35
Map resolution, global FSC 0.143 (Å) unmasked/masked	3.8/3.8	3.8/4.0	3.5/3.5	4.1/4.1
**Refinement**				
Initial model used for ECD (PDB code)	AlphaFold2 *Hs*PepT2	AlphaFold2 *Hs*PepT1	AlphaFold2 *Hs*PepT1	AlphaFold2 *Hs*PepT1
Model resolution (Å)				
FSC 0.5, masked/unmasked	3.9/4.0	4.1/4.3	3.7/3.8	4.2/4.4
FSC 0.143, masked/unmasked	3.7/3.8	3.8/3.9	3.5/3.6	4.0/4.1
Model composition				
Non-hydrogen atoms	5224	5147	5164	5030
Protein residues	659	657	659	643
ADP B factor (Å^2) mean	70.59	106.64	110.68	69.49
R.m.s deviations				
Bond lengths (Å) (#>4σ)	0.002 (0)	0.002 (0)	0.003 (0)	0.002 (0)
Bond angles (°) (#>4σ)	0.470 (1)	0.568 (1)	0.536 (0)	0.440 (0)
Validation				
MolProbity score	1.40	1.48	1.79	1.57
Clashscore	4.86	6.17	6.24	7.69
Rotamer outliers (%)	1.40	0.00	2.13	0.91
Ramachadran plot				
Favored (%)	97.86	97.23	96.76	97.16
Allowed (%)	1.98	2.77	3.24	2.84
Outliers (%)	0.15	0.00	0.00	0.00

Movie S1. Conformational changes of the SLC15 transporter family

Video morphing between the apo outward facing, substrate bound outward facing, substrate bound outward facing partially occluded, substrate bound inward facing partially occluded and apo inward facing PepT1 conformations as viewed parallel to the membrane. The outward facing conformations are based on the *Hs*PepT1 structures, the substrate bound inward facing partially occluded state was modeled on the *Hs*PepT2 structure and the apo inward facing conformation represents the AlphaFold model. The substrate has been omitted for clarity.
